# History of a prolific family: the *Hes/Hey*-related genes of the annelid *Platynereis*

**DOI:** 10.1186/2041-9139-5-29

**Published:** 2014-09-05

**Authors:** Eve Gazave, Aurélien Guillou, Guillaume Balavoine

**Affiliations:** 1 Institut Jacques Monod, CNRS, UMR 7592, CNRS/Université Paris Diderot-Paris 7, 15 rue H. Brion, Paris cedex 13 75205, France

**Keywords:** Hes superfamily, *Platynereis*, Nervous system, Segmentation, Chaetogenesis, DDC, Subfunctionalisation, Neofunctionalisation, Notch

## Abstract

**Background:**

The Hes superfamily or *Hes/Hey*-related genes encompass a variety of metazoan-specific bHLH genes, with somewhat fuzzy phylogenetic relationships. Hes superfamily members are involved in a variety of major developmental mechanisms in metazoans, notably in neurogenesis and segmentation processes, in which they often act as direct effector genes of the Notch signaling pathway.

**Results:**

We have investigated the molecular and functional evolution of the Hes superfamily in metazoans using the lophotrochozoan *Platynereis dumerilii* as model. Our phylogenetic analyses of more than 200 Metazoan *Hes/Hey*-related genes revealed the presence of five families, three of them (Hes, Hey and Helt) being pan-metazoan. Those families were likely composed of a unique representative in the last common metazoan ancestor. The evolution of the Hes family was shaped by many independent lineage specific tandem duplication events. The expression patterns of 13 of the 15 *Hes/Hey*-related genes in *Platynereis* indicate a broad functional diversification. Nevertheless, a majority of these genes are involved in two crucial developmental processes in annelids: neurogenesis and segmentation, resembling functions highlighted in other animal models.

**Conclusions:**

Combining phylogenetic and expression data, our study suggests an unusual evolutionary history for the Hes superfamily. An ancestral multifunctional annelid *Hes* gene may have undergone multiples rounds of duplication-degeneration-complementation processes in the lineage leading to *Platynereis*, each gene copies ensuring their maintenance in the genome by subfunctionalisation. Similar but independent waves of duplications are at the origin of the multiplicity of *Hes* genes in other metazoan lineages.

## Background

The basic helix-loop-helix (bHLH) protein superfamily comprises an ancient class of eukaryotic transcription factors (TFs) that are found in fungi, plants and metazoans [1]. These TFs are defined by the presence of a bHLH domain that is, a DNA-binding basic region (b) followed by two α-helices separated by a variable loop region (HLH), that serves as a dimerization domain and as a platform for protein interactions [2]. The bHLH superfamily is considered to be subdivided into 6 higher-order groups (named A to F) composed of evolutionarily related families of orthologous genes that share structural and biochemical properties. Among them, the *Hes* (*Hairy/enhancer of Split*) and the *Hey* (*Hairy/Enhancer of Split related with YRPW motif*) genes belong to two closely related families among the group E [2], and possess an additional protein-protein interaction domain, the Orange domain required for their function as transcriptional regulators [3]. Another molecular property of the HES/HEY proteins is the presence of a C-terminal tetrapeptide motif (WRPW or YRPW), which is known for HES proteins to recruit co-repressors of the *groucho*/*TLE1-4* family [4,5]. The Hes and Hey families include the well-known HAIRY, HAIRY-related, ENHANCER OF SPLIT proteins of *Drosophila* and the numerous mammalian HES and HEY proteins, as well as several other related proteins such as HERP, HEYL, HELT, HESL, DEC1, and DEC2 whose mutual relationships and relationships with HES and HEY proteins are still poorly understood [5-9]. This lack of knowledge often results in a confusing, non-consensual nomenclature of these genes.

These HES/HEY-related proteins are involved in a broad variety of molecular and developmental mechanisms across metazoans. They function as DNA-binding transcriptional repressors that control cell fate decisions in several contexts. These proteins are often, but not always, found as direct effector genes of the Notch signaling pathway [10-13]. This pathway, a direct juxtacrine signaling system, is involved in the control of cell identity, proliferation, differentiation and apoptosis in animals (see reviews [14-19]).

In deuterostomes and ecdysozoans, *Hes*/*Hey-*related genes are involved in crucial developmental events, in particular nervous system (NS) patterning and segment formation [20,21]. In mammalians, for example, *Hes* genes (notably *Hes1, Hes3* and *Hes5*) play an essential role in neural development by regulating proliferation, differentiation and specification of neural stem cells in both Notch-dependent and -independent manners [12]. These genes are also involved in regulating the maintenance of boundaries, which partition the NS into many compartments in a Notch-independent way [12,22]. Still in mouse, another *Hes*-like member, *HeyL* promotes neuronal differentiation of brain neural progenitor cells through the control of the BMP signaling, [23]. *Hes7* and *Hes1* are also key elements of the mouse molecular clock that, through the control of Notch, induce somite formation and are periodically expressed in anterograde wave-like fashion in the presomitic mesoderm (PSM), each wave leading to the generation of a pair of somites [22,24-26]. Other roles of *Hes* genes in mouse have been evidenced, such as regulating the maintenance of stem cells in digestive organs [12], the development of sensory organs (eye, inner ear) [5] and a critical role of *Hey* genes (*Hey1, Hey2, HeyL*) in the development of the cardiovascular system [5,27] in a Notch-dependent manner. Similar roles for *Hes/Her/Hey* genes in zebrafish and chick have been documented [12,25,26].

In *Drosophila,* the *Hairy* gene is involved in segmentation, during which it acts as a primary pair-rule gene required for the establishment of segments [28] but it also helps in defining the pattern of sensory bristles by repressing the formation of sense organ precursors [29]*,* in a Notch-independent way in both cases [28-30]. In contrast, the genes of the *Enhancer of split* (*Espl)* complex mediate the effects of Notch signaling in a process named lateral inhibition, during embryonic and adult neurogenesis. Activation of the *Espl* genes (except *m1*) blocks the accumulation of large amounts of proneural protein in most cells of the proneural clusters, preventing them from adopting a neural fate [31,32]. The *Hes* family gene *deadpan*, have been shown to regulate the self-renewal and specification of *Drosophila* neural stem cells, and to be involved in sex determination, both independently of Notch [33,34]. *Drosophila Hey* participates in alternative neuronal fate establishment during asymmetric divisions, both in a Notch-dependent and -independent manner [35]. In long germ-band arthropods, such as the spider *Cupiennius salei*[36,37], the myriapod *Strigamia maritima*[38], and the cockroach *Periplaneta americana*[39], some *Hairy-*related genes are expressed in segmental patterns through the control of Notch signaling, suggesting a role in the segmentation process, while in the short germ-band *Tribolium castaneum* a Notch-independent expression of *Hairy* is observed [40,41]. In the nematode *Caenorhabditis elegans*, a unique gene closely related to *Hes/Hey*, named *lin-22* was reported to be involved in patterning the peripheral NS (PNS), in a Notch-independent manner [42]. In addition, the members of the *Ref-1* family that encode unusual proteins containing two distinct bHLH domains, may be very divergent relatives of *Hes/Hey* genes and mainly mediate Notch signaling in various developmental processes, although Notch-independent expressions are also observed [43].

In lophotrochozoans, a major clade of protostomes often neglected in evolutionary developmental biology studies, the few data on *Hes* genes available so far mainly come from annelids. In the leech *Helobdella robusta*, *Hes* gene is expressed in the stem and progenitor cells (teloblasts and blast cells) of the posterior addition zone [44], under the control of Notch, and may be implicated in posterior elongation and segment formation [45,46]. In the polychaete *Capitella teleta*, three *Hes* genes have been shown to be expressed in a variety of embryonic territories. They are possibly regulated by Notch-dependent and -independent mechanisms, depending on the expression territories concerned [47]. The three genes are expressed in the posterior addition zone of the juvenile worm, which is responsible for the addition of segments. Two of the genes, *Cte-Hes2* and *3,* are also expressed in the brain and in the elongated trunk and *Cte-Hes2* is, in addition, expressed in the presumptive chaetal sacs, at the origin of the chaetae of the appendages, suggesting a role for *Hes* genes in neurogenesis, chaetogenesis and segmentation [47].

In non bilaterian metazoans, the roles of *Hes* genes have only been explored in cnidarians. In the anthozoan *Nematostella vectensis*, seven *Hes/Hey-like* genes have been reported to be expressed in a variety of territories, with distinct expression domains whose union seems to recapitulate the expression of the Notch receptor [48]. Blocking Notch signaling using small molecule inhibitors suggests that four of the *Hes* genes are targets of Notch signalling and are involved in cnidogenesis and neurogenesis [48]. Studies in adults and during budding of the hydrozoan *Hydra* suggest that Notch has a role in germ and nematocyte cell differentiation [49], as well as in boundary formation in the forming bud, via the regulation of the expression of *HyHes* (the only *Hes* reported in *Hydra*) [50]. In demosponges (Porifera), one *Hey* gene was identified in the genome of *Amphimedon queenslandica*[2] and recently, *Hes* genes were reported (by blast searches only) in the transcriptomes of two others demosponges (but without phylogenetic analyses confirming their assignment [51]).

In addition to the aforementioned studies aimed at defining the expressions and functions of *Hes* and *Hey* genes, there have been several analyses describing the genomic repertoire of these genes in various animals. These studies have shown a surprisingly variable number of *Hes/Hey* genes in these species and have suggested the occurrence of species- or lineage-specific duplications, for example, in the cnidarian *Nematostella vectensis*[6], the fruitfly *Drosophila melanogaster*[7], and the amphioxus *Branchiostoma floridae*[52,53]. Attempts to resolve the evolutionary relationships among *Hes/Hey* families members have so far focused on vertebrates [6,11] or insects [7]. A recent survey of *Hes/Hey* genes in 17 metazoan species (mainly vertebrates, plus 2 non-bilaterian species) has led authors to suggest that *Hey* genes were already present in the last common ancestor of metazoans, whereas *Hes* genes would have arisen in the stem lineage of Eumetazoans [2,6]. The authors of this study proposed a scenario with two rounds of expansions of this gene family, in the common ancestor of animals and vertebrates, respectively [6]. All these studies were, however, hampered by a dataset of taxa that are poorly representative of the metazoan diversity and by poor statistical support of phylogenetic trees [6]. Whereas the *Hes* and *Hey* genes are robustly separated into distinct clades, relationships among *Hes* genes are poorly resolved. A number of vertebrate sub-classes have been proposed recently and named *HesL, DEC1/2, Hes1/4, Hes2, Hes3, Hes5, Hes6, Hes7*[6] but it is unclear whether any of these sub-classes arose before the separation of the vertebrate lineage.

In this paper, we try to unravel several issues concerning the molecular evolution and functions of the *Hes/Hey*-related genes. (i) When and how did the multiplicity of *Hes/Hey*-related genes arise in the metazoan tree and how many families can be defined among them? (ii) Why have so many copies of *Hes/Hey*-related genes been conserved in the course of evolution? (iii) When and how have the multiple functions of *Hes/Hey*-related genes been acquired during metazoan evolution?

To gain insights into these questions, we studied the *Hes, Hey* and their related genes in the lophotrochozoan *Platynereis dumerilii.* Over the past decade, the annelid *Platynereis* has become a valuable model for evolutionary developmental biology studies. Importantly a number of comparative genomic studies have suggested that *Platynereis* is descending from a slow-evolving lineage and has therefore retained many ancestral bilaterian features including the ancestral composition of multigene families [54-57]. We identified a large family of 15 *Hes/Hey-*related genes in *Platynereis.* To determine whether these numerous *Platynereis* genes represent an ancestral bilaterian gene family or an independent gene radiation in the annelid lineage, we investigated broadly the origin and evolution of *Hes/Hey* genes and their distinct sub-families in animals. As a clear improvement compared to earlier studies, we sampled extensively animal lineages that branch outside bilaterians (cnidarians, ctenophores, sponges) to decipher the early steps of the family evolution in metazoans. We also sampled several lophotrochozoan species genomes, a bilaterian branch often neglected in phylogenomic studies. Our detailed phylogenetic analyses of more than 200 HES/HEY-related proteins show that three subfamilies (Hes, Hey and Helt) are pan-metazoan whereas two others seem to be restricted to protostomes (Stich) and chordates (Dec). Phylogenetic as well as genetic linkages analyses support the hypothesis of multiple independent *Hes* tandem duplications in almost each metazoan phylum, including in the *Platynereis* lineage. To test whether related *Platynereis* genes in the tree share similar expression patterns during embryogenesis; we determined the expression patterns through embryonic/larval development as well as during juvenile posterior elongation. We show that these genes are expressed in a wide variety of expression domains, (that is, mesodermal tissues, segments, NS). We discuss the possibility that *Platynereis Hes/Hey-*related genes, after duplication from a single ancestor, underwent a process of divergence by either neofunctionalization, that is, the random acquisition of a new function in the course of the accumulation of neutral mutation in duplicated genes [58] or subfunctionalization via the duplication-degeneration-complementation (DDC) model [58-61]. In the latter, it was postulated that degenerate mutations affect the gene functions, rendering neither copy alone sufficient to perform the ancestral functions and resulting in the partitioning of these ancestral functions in each paralogous copy [62].

## Methods

### Animal culture and collection

*Platynereis* embryos and juveniles were obtained from a breeding culture established in the Institut Jacques Monod (Paris), according to the protocol of Dorresteijn *et al*. [63]. Staging of the embryos was done following Fischer *et al*. [64]. Posterior parts of atokous worms regenerated 11 days after caudal amputation were obtained as previously described [65]. Embryos and larvae, as well as atokous worms 11 days after caudal amputation were fixed in 4% paraformaldehyde (PFA), 1 × PBS, 0.1% Tween20 and stored at -20°C in methanol 100% [66].

### Survey of *Platynereis dumerilii Hes/Hey*-related genes: identification, intron positions and cloning

*Platynereis Hes/Hey-*related genes were identified by sequence similarity searches against large collections of expressed sequence tags (ESTs) and genomic sequences (*Platynereis* resources, 4dx.embl.de/platy/, D Arendt, personal communication) [56] using *Drosophila* and/or vertebrate genes as query. Complete coding sequences were assembled from EST fragments using CodonCode Aligner (CodonCode Corporation, USA). For each *Platynereis* gene, putative exons positions were mapped on genomic DNA by comparison with ESTs using Artemis [67]. Large gene fragments were subsequently cloned by PCR using sequence-specific primers on cDNAs from mixed larval stages (primer sequences and PCR conditions are available upon request). PCR products were TA cloned into the PCR2.1 vector following the manufacturer’s instructions (Invitrogen, France) and sequenced. Partial cDNA obtained were then used as templates to produce RNA antisense probes for whole-mount *in situ* hybridization (WMISH) using Roche (France) reagents. Orthology relationships were defined using as criteria sequence similarities, presence of specific domains and phylogenetic analyses (see below). The fifteen newly identified *Platynereis* genes sequences were deposited in Genbank [KC999039 to KC999053].

### Sequences analyses

#### Data sources, sequence retrieving and domains composition

*Hes/Hey* gene searches were carried out using the tblastn or blastp algorithms [68] implemented in ngKlast (Korilog V 4.0, Questembert, France) with *Drosophila*, vertebrate and *Nematostella* proteins as query sequences, with the default BLAST parameters and a low cutoff E-value threshold of 0.1, against 24 genome datasets. Lists of BLAST hits were then reciprocally BLASTed against the human proteins dataset of the NCBI database to extract sequences related to the *Hes/Hey* family (reciprocal best hits [69]). Those genomes correspond to 24 metazoan species representatives of the main lineages of animals: Porifera, Ctenophora, Cnidaria, Placozoa, Lophotrochozoa, Ecdysozoa and Deuterostomia. For each species, we screened the genome assembly, the predicted protein sequence dataset and transcriptomes when available. Concerning the sponges, we concatenated a chimeric dataset from two *Oscarella* species: *Oscarella carmela* from which the genome is accessible and an undescribed *Oscarella* specimen (*Oscarella* sp.) from which only EST were available. Indeed, the *Hes* repertory of *Oscarella carmela* lacks several representatives that were present in the EST dataset of another *Oscarella* and their addition are critical to understand the origin of the *Hes* family.

The presence of the *Hes/Hey*-related specific protein domains (that is, bHLH, Orange and WRPW peptides) was systematically checked by scanning sequences with both NCBI Conserved Domain search option V3.10 [70] and InterProscan V.42 online software [71]. An important proportion of the *Hes* predicted sequences (1/4 roughly) do not harbor an Orange domain. We tried to ensure that these Orange domains are genuinely missing by checking predicted sequences against genomic scaffolds and screening specifically for Orange sequences. However, in the absence of exhaustive transcriptome data in some species, we cannot exclude that in a limited number of cases, the lack of an Orange domain results from faulty sequence prediction. Last, a complete list of genomic scaffolds carrying the predicted sequences was produced for each species and the presence of genomic clusters was established for a number of them.

#### Phylogenetic analyses

The predicted amino-acid sequences of the identified *Platynereis* gene fragments were aligned with their presumptive orthologs from 24 metazoan species. Two group-B bHLH members: sterol regulatory element-binding protein (SREBP) and microphthalmia-associated transcription factor (Mitf) were selected as the outgroup in order to root the *Hes/Hey-*related tree. Only the bHLH domains of those sequences were included and aligned for two species: *Danio rerio* and *Lottia gigantea*. Alignments were performed with MUSCLE 3.7 online [72,73] under default parameters and adjusted manually in Bioedit [74]. Only parts of the alignments corresponding to the bHLH, Orange and WRPW peptides, when presents (112 amino acids altogether) were used for the phylogenetic analyses. Two datasets were used for the analyses, one including all sequences for all species (n = 201) and the other containing only sequences where both domains (bHLH and Orange) were identified (n = 154). The phylogenetic trees were constructed using two different approaches: the maximum likelihood (ML) and the Bayesian analyses. ML analyses were performed with the PHYML 3.0 program under an LG model of amino acid substitution [75], a model that was shown to be the most efficient. To take into account rate variation among sites, we computed likelihood values by using an estimated gamma law with six substitution rate categories and we let the program evaluate the proportion of invariant sites. Statistical support for the different nodes was assessed by both the approximate likelihood ratio test (aLRT) [76] and bootstrap (BP) analysis [77] with 500 replicates. Bayesian analysis was performed with MrBayes 3.2.1 [78], using the WAG fixed model, as the LG model is not available. Two sets of six independent simultaneous metropolis-couples Markov chains Monte Carlo were run for 10 and 20 million generations (for the restricted and all inclusive alignments respectively) and sampled every 500^th^ generation. We estimated that convergence was obtained if the average standard deviation of split frequencies reached a threshold value of 0.05. The trees obtained were mixed and an adequate burn-in was removed (above 25% of tree and parameters). Bayesian posterior probabilities (PP) were used for assessing the confidence value of each node [79]. Phylogenetic trees were visualized, rooted and edited using FigTree V.1.4.0 [80]. The tree topologies showed are from the ML analysis and all nodes, even moderately supported ones, were conserved because taxa number and composition, in addition to statistical support, are keys to discussing the validity of nodes in such a broad phylogenetic analysis.

We also performed parsimony reconstruction of character evolution based on a consensus Metazoan phylogenetic tree using Mesquite software version 2.72 [81]. Character used in those analyses is the number of gene per species that was encoded in a character matrix. Analyses were performed for Hes and Hey family as well as more than 40 other bHLH families, based on previously published datasets [2]. Sampling of the precedent paper differs slightly from this study, implying the presence of missing data in the character matrix.

### Visualization of *Platynereis HES* expression patterns by whole mount *in situ* hybridization

Single NBT/BCIP whole-mount *in situ* hybridization was performed as previously described [66] on five larval stages (24, 33, 48, 55 and 72 h post fertilization (hpf)) and during post-embryonic posterior elongation. For the latter, we performed WMISH on worms 11 days after posterior amputation as post-caudal regeneration posterior elongation is a proxy to normal posterior elongation [82]. Bright-field images were taken on a Leica microscope. Adjustments of brightness, contrast and Z projections were performed using the ImageJ and Photoshop software.

## Results and discussion

### Origin and evolution of the Hes superfamily

#### The Hes superfamily in *Platynereis*

**Figure 1:**
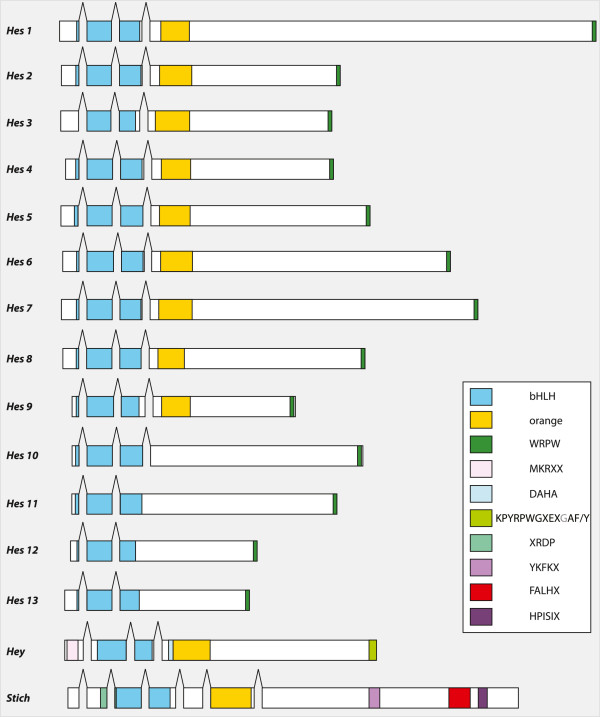
***Platynereis Hes/Hey*-related genes structures and domains.** The intron positions, indicated by gaps, are essentially conserved in 10 *Pdu-Hes* (1 to 10). The different domains are schematized by colored boxes (see inset). Newly defined domains (see text for details and Figure 4) for *Pdu-Hey* and *Pdu-Stich* are also mentioned. All but four *Platynereis* genes possess both the bHLH and the Orange domains. All but *Pdu-stich* have a WRPW/YRPW C-terminal motif.

Exhaustive searches on the genome of *Platynereis* complemented with several EST datasets led us to identify no less than 15 *Hes/Hey*-related genes coding for proteins of various lengths: from 215 to 642 amino-acids. While all of them possess the conventional bHLH domain, four genes (*Pdu-Hes10; Pdu-Hes11; Pdu-Hes12; Pdu-Hes13*) lack the Orange domain (Figure 1). These absences represent presumably secondary evolutionary losses of an ancestral Orange domain although we cannot exclude the possibility of a non-perfect assembly of the genome that could impair our domain predictions. All but *Pdu-Stich* possess the WRPW terminal domain, modified in WQPW in *Pdu-Hes9* and in YRPW in *Pdu-Hey*.

In *Pdu-Hes1* to *Pdu-Hes10*, a conserved pattern of intron positions is observed, two of them being located at exactly homologous positions in the bHLH coding sequence, while the third one is situated between bHLH and Orange coding sequences (Figure 1). In *Pdu-Hes11, Pdu-Hes12* and *Pdu-Hes13*, lacking the Orange domain, only the first two introns (in the bHLH domain) are found, whereas *Pdu-Hey* and *Pdu-Stich* harbor only the second homologous intron (Figure 1). *Pdu-Hey* and *Pdu-Stich* are peculiar with respectively three and six introns, only one of which is in shared positions with other *Platynereis Hes*-related genes.

This high number of *Hes/Hey*-related gene copies in the *Platynereis* genome is somewhat surprising, given the evolutionary conservatism displayed in other gene families such as the *Wnt*[57], *Hox*[83] and bHLH [2] genes. A number of other metazoans share a high number of *Hes*-related genes. This assessment led us to question the evolutionary origin of such diversity, to shed light on this issue and prompted us to extend our genomic analyses to the scale of the whole metazoan clade.

#### The Hes superfamily in Metazoa consists of three pan-metazoan families: Hes, Hey and Helt

We performed a detailed search of *Hes*/*Hey*-related genes in metazoan species representatives of all main metazoan lineages (that is Deuterostomia, Ecdysozoa, Lophotrochozoa, Ctenophora, Cnidaria, Placozoa and Porifera). Details of species used, genomic resources access, sequences names, domains presence or absence as well as scaffold/chromosomes numbers where the sequences are located (when available), are presented in the Additional file 1. We especially surveyed lophotrochozoan and non-bilaterian species as they have been neglected in earlier studies and are especially informative on bilaterian, eumetazoan and metazoan ancestral states, respectively.

**Figure 2:**
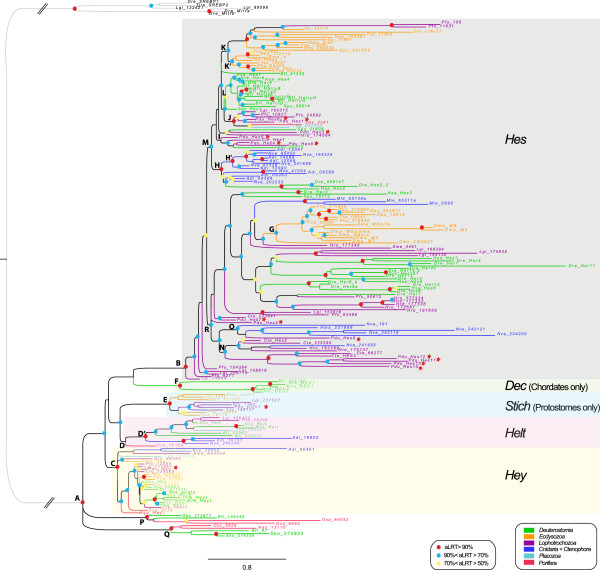
**Phylogenetic tree of *Hes/Hey*-related genes based on the complete dataset.** The tree topology is obtained from the ML analysis and rooted on *SREBP* and *Mitfa* bHLH genes. A color code was used for nodes robustness (see inset). Branch and sequence names have specific colors following a color code based on the clade/phylum they belong to (see inset). The letters in front of the nodes refer to Table 1. Dashed lines indicate that branches were artificially reduced for a more aesthetic representation. Hes, Dec, Stich, Helt and Hey family members are highlighted by a specifically colored background.

**Figure 3:**
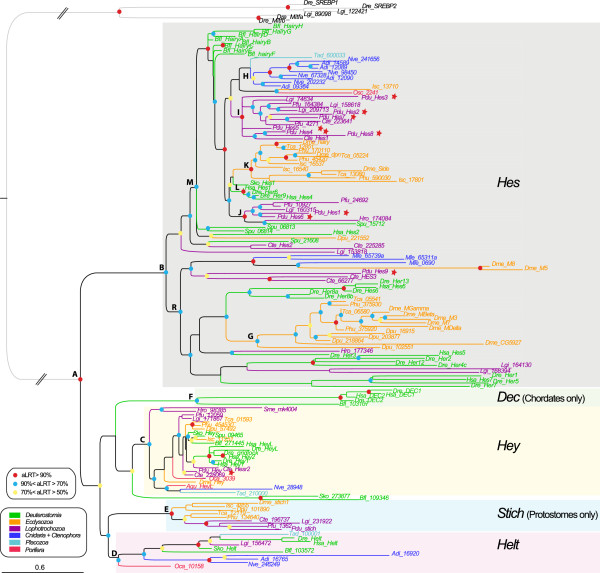
**Phylogenetic tree of *Hes/Hey*-related genes based on the partial dataset.** Partial dataset include all sequences that possess both the basic helix-loop-helix (bHLH) and the Orange domains. The tree topology is obtained from the maximum likelihood (ML) analysis and rooted on Sterol regulatory element binding protein (*SREBP*) and microphthalmia-associated transcription factor (*Mitfa*) bHLH genes. A color code was used for node robustness (see inset). Branch and sequence names have specific colors following a color code based on the clade/phylum they belong to (see inset). The letters in front of the nodes refer to Table 2. Dashed lines indicate that branches were artificially reduced for a more aesthetic representation. Hes, Dec, Stich, Helt and Hey family members are highlighted by a specifically colored background.

In a first approach, we built ML and bayesian trees of the complete dataset, that is including those sequences for which no Orange domain was found (Figure 2, Table 1). As the evolution of the Hes/Hey family is rather complex, we first assessed how many strongly supported pan-metazoan clades are evidenced by these trees. These clades reflect the existence of a number of ancestral genes that were present in a metazoan ancestor and are evidenced by highly supported clades (aLRT >90%) in which a majority of metazoan phyla are represented. Only three mutually exclusive clades of this nature exist in the complete tree: nodes C, D’ and B, corresponding respectively to Hey, Helt and a large clade grouping most remaining *Hes* genes. Strikingly, the emergence of these three clades predate the last common ancestor of all metazoans as genes belonging to each of them are found in three (of the four) non-bilaterian phyla considered here, the sponges, the placozoan and the cnidarians (Figures 2 and 3, Tables 1 and 2) in the hypothesis of sponges being the sister group to all other metazoans species [84]. Recently an alternate view of the relationships of non bilaterian phylum have emerged and some authors considered that ctenophore are indeed the sister group to all others metazoans [85]. In the case of the Hes superfamily, *Mnemiopsis Hes/Hey*-related genes repertory is especially poor, with only three long-branch *Hes* genes, and less informative compared to sponges.

**Table 1:** Summary of topologies and node supports obtained with the Hes superfamily complete dataset analyses

**Node names**	**Node descriptions**	**Complete dataset**			**Comments**		
		**ML - aLRT**	**ML - bootstrap**	**Bayes - PP**	**ML - aLRT**	**ML - bootstrap**	**Bayes - PP**
**A**	*Hes + Dec + Hey + Stich + Helt*	0,99	86	1	9 sequences are not included in any family	9 sequences are not included in any family	9 sequences are not included in any family
**B**	*Hes*	0,98	22	0,91			
**C**	*Hey*	0,92	45	0,95			
**D**	*Helt*	0,15	20	0,78			*Oca_10158* is not included in *Helt*
**D'**	*Helt* subgroup	0,9	30	0,78	*Oca_10158* is excluded	*Oca_10158* is excluded	
**E**	*Stich*	1	92	0,94			
**F**	*DEC*	0,92	100	0,86			
**G**	*E(Spl)*	0,73	19	0,96	15 sequences of ecdysozoans	15 sequences of ecdysozoans	15 sequences of ecdysozoans
**H**	Cnidarian *Hes* group 1	0,9	0	0,57	13 sequences + 3 sequences of deuterostomian	13 sequences + 3 sequences of deuterostomian	6 sequences only
**H'**	Cnidarian *Hes* subgroup of group 1	0,92	86	0,57	8 sequences	8 sequences	6 sequences only
**I**	Lophotrochozoan *Hes* group 1	0,44	2	_	4 sequences + *Spu_06813*	4 sequences + *Spu_06813*	Not found
**J**	Lophotrochozoan *Hes* group 2	0,6	3	0,61	5 sequences	5 sequences	5 sequences + *Spu_15712*
**K**	Ecdysozoan *Hes* group 1	0,84	0	_	17 sequences including *Dme_Hairy, side* and *dp, Cel_Lin-22* + 2 lophotrochozoan sequences	17 sequences including *Dme_Hairy, side* and *dpn Cel_Lin-22* + 2 lophotrochozoan sequences	Not found
**K'**	Ecdysozoan *Hes* subgroup of group 1	0,92	0	_	8 sequences including *Dme_Hairy* and *dpn*	8 sequences including *Dme_Hairy* and *dpn*	Not found
**L**	Deuterostomian *Hes* group 1	0,8	5	_	14 sequences	14 sequences	Not found
**M**	*Hes* subgroup	0,84	0	_	65 sequences including Lophotrochozoan *Hes* groups1 and 2, Ecdysozoan *Hes* group 1,	65 sequences including Lophotrochozoan *Hes* groups1 and 2, Ecdysozoan *Hes* group 1,	Not found
					Cnidarian *Hes* group 1 and Deuterostomian *Hes* group 1	Cnidarian *Hes* group 1 and Deuterostomian *Hes* group 1	
**N**	Lophotrochozoan *Hes* group 3	0,71	1	1	10 sequences + 1 sequence of *Nematostella*	10 sequences + 1 sequence of *Nematostella*	6 sequences only
**O**	Cnidarian *Hes* group 2	0,81	18	_	5 sequences + 1 sequence of *Platynereis*	5 sequences + 1 sequence of *Platynereis*	Not found
**P**	Sponges + deuterostomes group 1	0,99	71	1	4 sequences (2 sponges + 2 deuterostomes)	4 sequences (2 sponges + 2 deuterostomes)	4 sequences (2 sponges + 2 deuterostomes)
**Q**	Sponges + deuterostomes group 2	0,96	89	1	5 sequences (2 sponges + 3 deuterostomes)	5 sequences (2 sponges + 3 deuterostomes)	5 sequences (2 sponges + 3 deuterostomes)
**R**	Hes subgroup 2	0,87	0	_	70 sequences including E(spl), Lophotrochozoan Hes group 3 and Cnidarian Hes group 2	70 sequences including E(spl), Lophotrochozoan Hes group 3 and Cnidarian Hes group 2	Not found

*Hes/Hey*-related genes complete dataset was analyzed both with maximum likelihood (ML) and Bayesian algorithms. Node supports were tested by approximate likelihood ratio test (aLRT) (0 to 1) and bootstraps (0 to 100) for ML, and with posterior probabilities (PP, 0 to 1) for Bayes; results were provided in this table. Nodes names and descriptions correspond to the nodes of the Figure 2. Comments on clade for all analyses are provided, when relevant.

**Table 2:** Summary of topologies and nodes supports obtained with the Hes superfamily partial dataset analyses

**Node ** **names**	**Node descriptions**	**Partial dataset**			**Comments**		
		**ML - aLRT**	**ML - bootstrap**	**Bayes - PP**	**ML - aLRT**	**ML - bootstrap**	**Bayes - PP**
**A**	*Hes + Dec + Hey + Stich + Helt*	0,99	96	1	2 sequences are not included in any family :	2 sequences are not included in any family :	*Oca_10158* is included in
					*sko_273877* and *Bfl_109346*	*sko_273877* and *Bfl_109346*	no family
**B**	*Hes*	0,89	32	0,97			
**C**	*Hey*	0,86	44	0,97			*sko_273877* and *Bfl_109346*
							are included in Hey
**D**	*Helt*	0,72	28	0,95			*Oca_10158* is outside Helt
**E**	*Stich*	1	96	0,99			
**F**	*DEC*	0,87	60	0,92			
**G**	*E(Spl)*	0,8	4	0,8	*Dme-M8* and *Dme_M5* are not included	*Dme-M8* and *Dme_M5* are not included	*Dme-M8* and *Dme_M5* are included
**H**	Cnidarian *Hes*	0,69	55	0,69	8 sequences	8 sequences	8 sequences
**I**	Lophotrochozoan *Hes* group 1	0,9	1	_	13 sequences	13 sequences	Not found
**J**	Lophotrochozoan *Hes* group 2	0,92	6	0,78	6 sequences + *Spu-15712*	6 sequences + *Spu-15712*	5 sequences + *Spu-15712*
**K**	Ecdysozoan *Hes* group 1	0,93	1	_	12 sequences including *Dme_Hairy, side* and *dpn*	12 sequences including *Dme_Hairy, side* and *dpn*	Not found
**L**	Deuterostomian *Hes* group 1	0,97	58	0,98	4 sequences of *Danio* and *Homo*	4 sequences of *Danio* and *Homo*	4 sequences of *Danio* and *Homo*
**M**	*Hes* subgroup	0,91	0	_	48 sequences including Lophotrochozoan *Hes*	48 sequences including Lophotrochozoan *Hes*	Not found
					groups 1 and 2, Ecdysozoan *Hes* group 1,	groups 1 and 2, Ecdysozoan *Hes* group 1,	
					Cnidarian *Hes* and Deuterostomian *Hes* group 1	Cnidarian *Hes* and Deuterostomian *Hes* group 1	
**R**	Hes subgroup 2	0,76	1	_	39 sequences including *E(spl)*	39 sequences including *E(spl)*	Not found

*Hes/Hey*-related genes partial dataset was analyzed both with maximum likelihood (ML) and Bayesian algorithms. Node supports were tested by approximate likelihood ratio test (aLRT) (0 to 1) and bootstraps (0 to 100) for ML, and with posterior probabilities (PP, 0-1) for Bayes; results were provided in this table. Node names and descriptions correspond to the nodes of Figure 3. Comments on clade for all analyses are provided, when relevant.

In addition to B, C and D’ groups, several smaller but well-supported clades show a more restricted taxonomic composition. Node E contains only protostome genes, both from ecdysozoan and spiralian taxa, and presumably reflects a new, previously unrecognized ancestral protostome gene related to *Drosophila Sticky ch1*[86]. Node F contains only vertebrate *Dec* genes grouped with an amphioxus gene, thus likely indicating an ancestral chordate *Dec* gene. Two remaining well supported clades (nodes P and Q) represent only small subsets of animal species (sponges, hemichordate and cephalochordate). In addition, these genes display long branches. Therefore, we consider that both nodes P and Q are unlikely to represent ancestral metazoan genes but are rather derived genes grouped together by artifact. While the monophyly of the Hes/Hey family as a whole is well-supported, relationships within four interphyletic clades (Hes, Hey, Helt and Stich) are poorly resolved (Figure 2, Table 1).

**Figure 4:**
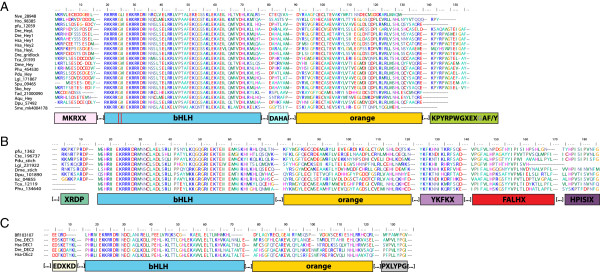
**Alignments of protein conserved motifs/domains for three Hes-related families. (A)** Hey family conserved motifs/domains. Above the basic helix-loop-helix (bHLH) and Orange domains, three conserved motifs/domains are proposed: MKRXX, DAHA, KPYRPWGXEXGAF/Y. Red lines indicate a specific glycine in the 6^th^ position of the bHLH domain that can be considered as a molecular signature of Hey family. Brackets around dots indicate that the alignments were artificially cut for a more aesthetic representation. **(B)** Stich family conserved motifs/domains. Above the bHLH and Orange domains, four new conserved motifs/domains are proposed: XRDP, YHFKX, FALHX, HPISIX. **(C)** Dec family conserved motifs/domains. Above the bHLH and Orange domains, two new conserved motifs/domains are proposed: EDXKD and PXLYPG.

One possible explanation why relationships between genes are so poorly resolved within the Hes clade could be the rapid evolution of the sequences of a large number of genes. Indeed, many genes coding for a protein with no Orange domain have a long branch especially within the R clade. We wanted therefore to test the possibility of assessing the phylogeny of the genes with a conserved protein domain structure (presence of the Orange domain) separately (Figure 3, Table 2). The resulting tree was not fundamentally different in its overall architecture from the complete dataset tree. In particular, nodes corresponding to the *Hey* (C), *Helt* (D), *Stich* (E), *Dec* (F) and *Hes* genes (B) were still present, although with slightly diminished statistical significance. Within *Hes* genes, a clade with relatively short branched genes (M) was present in both trees, with a large majority of the same genes. A clade with long branched genes (R) was also found with some statistical support. This clade is however much smaller than in the complete dataset tree because many proteins with no Orange domain were initially included in this group.Based on these phylogenetic results, we can redefine five families, also supported by specific additional proteic motifs (Figure 4). Three families are pan-metazoan (the Hes, Hey and Helt families) and two others are clade-specific (the Stich and Dec families).

The evolutionary history of the well-known Hes family was already investigated in a study mainly focused on vertebrates [6]. This recent study failed to evidence clear relationships among this large family outside vertebrates. Not surprisingly, we observed a similar fuzzy situation in our own analyses. Nevertheless, in opposition to precedent statement (based only on one sponge species, that is, *Amphimedon queenslandica*, Demospongiae) [6], we found the evidence of a real *Hes* gene from another sponge lineage, the Homoscleromorpha (recently nominated as the fourth sponge lineage [87]). Accordingly we also totally disagree with the idea of a primitive tetrapeptide FRPW, found in the *A. queenslandica Hey/1/2/L* gene, which could represent the ancestor of *Hes* WRPW domain. These points highlight the fact that a unique representative species of a large phylum is not sufficient and can lead to erroneous evolutionary interpretations.

The Hey family [2] is present in all metazoan lineages included in these analyses. This family is characterized in addition to bHLH and Orange domains by an N-terminal motif named MKRXX, while shorter than the motif 1 previously proposed [6], by an extended well-conserved C-terminal motif, 13 amino-acid-long, renamed KPYRPWGXEXGAF/Y and another short motif (7 aa) located between the bHLH and Orange domains named DAHA. Finally, we observed that a specific glycine is found only in *Hey* sequences, in the 6^th^ amino acid position of the bHLH domain and can be considered as a molecular signature of *Hey* (Figure 4A). Our trees are compatible with the presence of a single *Hey* gene in the last metazoan, eumetazoan and bilaterian ancestor and a single gene has been retained in many metazoan species.

The previously poorly-defined Helt family [6,88] encompasses 12 members of Deuterostomes, Lophotrochozoa, Cnidaria, Placozoa and Porifera but surprisingly no Ecdysozoa. This family named HESL in a previous study [6] was supposed to be composed of eumetazoan representatives only. The presence of sponge and placozoa sequences within this family rejected this hypothesis. This robust clade, in our phylogenetic analyses, is not supported by any discrete molecular signature. Nevertheless, intron numbers and positions are conserved in Placozoa, Cnidaria, Lophotrochozoa and Deuterostome representative species, except for *B. floridae* sequences. In others, the first intron is found just before the bHLH domain, the 2^nd^, inside the bHLH, and the third between the bHLH and the Orange domains. *Trichoplax* sequence harbors a supplementary intron in the Orange domain (data not shown). Trees are compatible with the existence of a single *Helt* gene in the last metazoan, eumetazoan and bilaterian ancestor. This *Helt* gene has been secondarily lost in an ecdysozoan ancestor as well as an annelid ancestor.

The new Stich (named after the fruit fly gene *Sticky ch1*) family forms a robust clade of protostomes sequences only, which has never been identified in previous studies [6]. Detailed analyses of those nine sequences revealed the presence of four specific conserved motifs shared by all sequences (except for the first one) in addition to the classical bHLH and Orange domains (Figure 4B). We named XRDP the first Stich-specific motif, 9 amino-acid long, and located just in front of the bHLH. This motif seems to be absent from two sequences (*Phu134640* and *Tca12119*) but as those sequences are incomplete in the N-terminal part (Figure 4B) we cannot exclude that their absence is due to an imperfect genome annotation. The second motif, named YKFKX is 14 aa long and is located between the Orange domain and the C-terminal part of the protein. The third Stich motif is longer (27 aa) and also located between the Orange domain and the C-terminal part of the protein. We named it FALHX while three sequences do not harbor exactly this motif (especially the *Pinctada* sequence). The fourth and last specific motif of the Stich family is located in the C-terminal part. Composed of 12 aa and named HPISIX, it is found in all sequences except the shorter *Lottia* sequence. Intron numbers and positions are not conserved among *Stich* sequences (data not shown). Trees are compatible with a single *Stich* gene having been present in the last protostome ancestor and a single gene is present in most of its extant protostome descendants.

The Dec family was already known and supposed to be composed of chordate representatives as well as a *Drosophila* sequence, although no phylogenetic data support this last point [6]. Furthermore, two diagnostic motifs named motifs 2 and 3 have been proposed by Zhou *et al*. [6]. Our phylogenetic analysis revealed that the Dec family is specific to chordates solely and while the motifs 1 and 2 are indeed found in the vertebrate members, they are clearly not conserved in the *Branchiostoma* sequence. We nevertheless found two short specific motifs of 9 aa, EDXKD and PXLYPG, respectively in the N-terminal and C-terminal parts of the proteins, that are diagnostic of Dec members (Figure 4C). Intron numbers and positions are almost totally conserved; with little variation for the *Branchiostoma* protein. Indeed, all of them have a first intron in the non-conserved N-terminal part of the protein, the 2^nd^ intron is found in the middle of the newly described EDXKD motif, and the 3^rd^ one is in the middle of the bHLH domain. For the chordate sequence, the 4^th^ intron is located between the bHLH and the Orange domain, while is it inside the Orange domain in the *Branchiostoma* protein (that also possesses a supplementary 5^th^ intron) (data not shown). One *Dec* gene was present in the last common ancestor of chordates.

The numbers of genes for each species in each gene clade reveals contrasting situations (Table 3). In a majority of metazoan species, a single gene was found in each species for the Hey, Helt and Stich clades. This is compatible with the hypothesis that a single gene was present in the metazoan (*Hey*, *Helt*) or protostome (*Stich*) last common ancestor. One exception is the presence of three *Hey* paralogues in vertebrates *Homo* and *Danio*, presumably the result of the double whole genome duplication (2R) postulated in a vertebrate ancestor. By contrast, the number of *Hes* genes is extremely variable, ranging from one single gene in the sponge *Oscarella*, the placozoan *Trichoplax*, the cnidarian *Hydra*, the deuterostome *Saccoglossus*, the ecdysozoan *Caenorhabditis*, and the spiralian *Schmidtea* to 11 in the cnidarian *Nematostella*, 11 in the ecdysozoan *Drosophila*, 13 in the spiralian *Platynereis* and no less than 22 in the deuterostome *Danio*. This indicates that the evolution of the Hes family in each of four big animal clades (cnidarians, spiralians, ecdysozoans and deuterostomes) has been complex with numerous independent gene duplications, or numerous gene losses, or a combination of both phenomena.

**Table 3:** **Classification of the 208 *Hes/Hey*-related sequences identified in 24 metazoan species**

**Species names**	**Number of sequences**						
	***Hes***	***Hey***	***Helt***	***Stich***	***Dec***	**Unknown**	**Total**
*Amphimedon queenslandica*	0	1	0	0	0	1	2
*Oscarella* chimeric	1	1	1	0	0	3	6
*Trichoplax adhaerens*	1	1	1	0	0	0	3
*Mnemiopsis leidyi*	3	0	0	0	0	0	3
*Nematostella vectensis*	11	1	1	0	0	0	13
*Acropora digitifera*	7	1	2	0	0	0	10
*Hydra magnipapillata*	1	0	0	0	0	0	1
*Capitella teleta*	6	2	0	1	0	0	9
*Lottia gigantea*	11	1	1	1	0	0	14
*Helobdella robusta*	9	1	0	0	0	0	10
*Platynereis dumerilii*	13	1	0	1	0	0	15
*Pinctada fucata*	8	1	1	1	0	0	11
*Schmidtea mediterranea*	1	1	0	0	0	0	2
*Daphnia pulex*	5	1	0	1	0	0	7
*Drosophila melanogaster*	11	1	0	1	0	0	13
*Pediculus humanus*	5	1	0	1	0	0	7
*Ixodes scapularis*	4	1	0	1	0	0	6
*Tribolium castaneum*	6	1	0	1	0	0	8
*Caenorhabditis elegans*	1	0	0	0	0	0	1
*Danio rerio*	22	3	1	0	2	0	28
*Homo sapiens*	7	3	1	0	2	0	13
*Strongylocentrotus purpuratus*	4	1	0	0	0	0	5
*Saccoglossus kowalewski*	1	1	1	0	0	3	6
*Branchiostoma floridae*	9	1	2	0	1	2	15
**Total**	**147**	**26**	**12**	**9**	**5**	**9**	**208**

All of them were assigned to a particular sub-family following phylogenetic analysis. Numbers of representatives for each sub-family for each species are detailed. Definitions of the sub-families are provided in the text.

Our exhaustive analyses of Hes superfamily in a broad variety of metazoan organisms, especially lophotrochozoans and non-bilaterians ones, allow us to grasp the early evolutionary history of this group. Indeed, our phylogenetic data, in opposition to precedent statements [6], clearly show the presence of three pan-metazoan families (Hes, Hey and Helt) that we inferred from the presence of indisputable *Hes*, *Hey* and *Helt* orthologs in sponge species, (considering that sponges are the sister group to all other metazoan species [84]). *Stich* members are specific to the protostomes indicating a likely appearance of the Stich family in the direct ancestry of this lineage. While this is less parsimonious, the Stich family could have been already present in *Urbilateria* (the bilaterian common ancestor) and lost in the deuterostomes. *Urbilateria* possessed at least 3 *Hes/Hey* related genes (*Hes, Hey* and *Helt*).

#### Multiple *Hes* gene independent duplications in many metazoans

The Hes family is composed of a high number of *Hes* sequences; with a great variability in the number of genes found in metazoan species (Table 3) from one in the enteropneust *Saccoglossus* to 22 in the vertebrate *Danio*; more than 60 of these genes are found in a clade of relatively short branched taxa (Figures 2 and 3, M node). Many more derived sequences, with longer branches are found in a second, poorly supported clade, R.

As already observed on a smaller scale [6], in both clades, genes tend to be grouped into lineage-specific clades. In the clade M, a big clade of ecdysozoan genes (K or K’), a large clade of deuterostome genes (L), two groups of lophotrochozoan genes (I and J) and robust clades of cnidarian genes (H and H’) are found. Sponge and Placozoa representatives are grouped together. Six of the *Platynereis Hes* genes: *Pdu-Hes1*, *Pdu-Hes3*, *Pdu-Hes4*, *Pdu-Hes5*, *Pdu-Hes6, Pdu-Hes8* are found in the lophotrochozoan clades I and J. The other part of *Hes* subfamily clade (poorly supported clade R) contains diverse divergent sequences notably the *Enhancer of split* complex members that are grouped together (nodes G, with two *Drosophila* sequences excluded in the partial dataset). Large cnidarians and lophotrochozoan-specific clades (nodes N and O) are also found, with four derived *Platynereis* sequences (*Pdu-Hes10* to *13)* within the latter*.*

**Figure 5:**
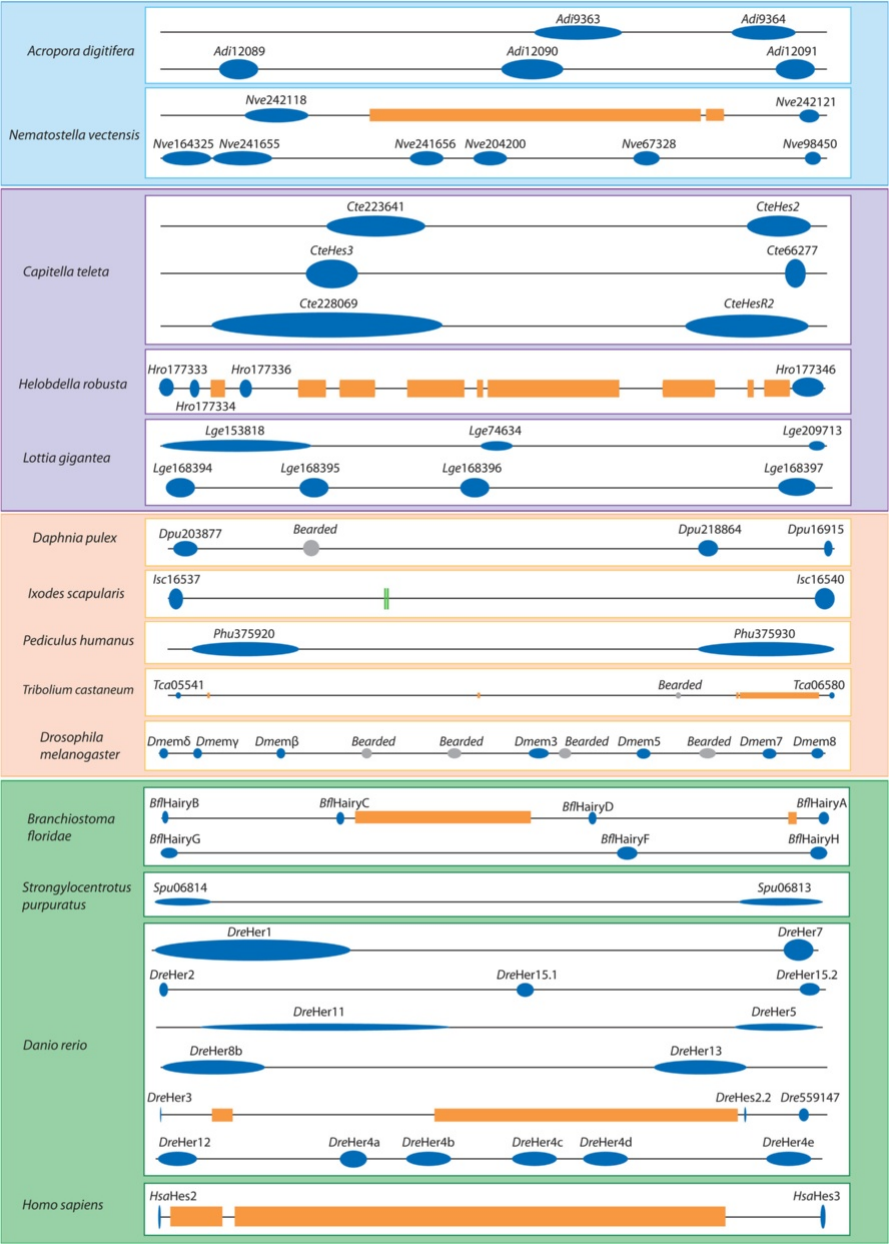
**Genomic clustering of the Hes superfamily genes in diverse metazoan representatives.** Blue dots correspond to *Hes/Hey*-related genes (name mentioned above); orange squares represent non-*Hes/Hey*-related intervening genes; grey dots indicate non-*Hes E(spl)* members (name mentioned above), green lines represent microRNA. Boxes follow the same color code as in phylogenetic trees (Figures 2 and 3): cnidarians are in blue, lophotrochozoans are in purple, ecdysozoans are in orange and deuterostomians are in green. Physical linkages between *Hes/Hey*-related genes are found in each of these major lineages, with two to six clustered genes.

Such phylogenetic relationships tend to indicate the presence of a limited number of ancestral genes and a large number of independent gene duplications in various lineages. Strong evidence of such gene duplications is the persistence of chromosomal gene linkages, indicative of tandem duplications. We checked chromosome locations and genetic linkages for species that present specific duplications and from which those data are available (that is, 2 Cnidaria, 3 Lophotrochozoa, 5 Ecdysozoa and 4 Deuterostomes) and have detailed the start and end positions of the genes in the scaffold as well as the gene strand, in Additional file 2. We found the presence of one or more clusters of *Hes* genes in all the 14 species genomes explored (Figure 5). In all but two species (*S. purpuratus* and *H. sapiens),* the phylogenetically related genes are also clustered and so physically linked. This situation is especially obvious in cnidarians where two clusters of two and three genes were found in *Acropora* and two clusters of two and six genes in *Nematostella*. For *Acropora,* all the clustered genes are phylogenetically related while only 6 on 8 *Nematostella* genes are. In *Capitella teleta,* three clusters were found and surprisingly one corresponds to *Hey* genes. This is the only case of a non-*Hes* tandem duplication. In the two other lophotrochozoans, several clusters of three and four genes were found. As already described, clustered genes are also found in the Ecdysozoans *E(Spl)* complex [7], in the amphioxus [52,53] and in zebrafish [6]. A sea urchin complex of two *Hes* genes that are not phylogenetically related was found, but the *Spu-21608* sequence placement in the tree among lophotrochozoa is doubtful. Another argument in favor of this hypothesis is provided by the parsimony reconstruction of the character number of *Hes* genes (based on a theoretical metazoan tree [84]) analysis (Additional file 3, A). From the observed pattern, we conclude in favor of the presence of one ancestral gene that underwent several lineage specific duplications. Gene losses may also have occurred but cannot be inferred precisely.

Can we thus reconstitute an ancestral number of *Hes* genes in the last metazoan, eumetazoan and bilaterian ancestors? Given the relatively low significance of phylogenetic resolution in the *Hes* clade, any proposal will remain tentative. We however propose that a single *Hes* gene may have been present in the metazoan ancestor and one or possibly two in the eumetazoan and bilaterian ancestors. These ancestral genes underwent numerous gene duplications in several, but not all, metazoan lineages (Additional file 3, A). The fact that species-specific clades exist also revealed that some of these duplications are recent, as highlighted in the zebrafish [6], amphioxus [53] but also in *Helobdella* and *Lottia*, and presumably *Platynereis* genomes*.* The presence of a single indisputable *Hes* gene*,* in the sponge *Oscarella*, embedded in the short branch clade M, is a clear new indication that the family originated in a metazoan ancestor. The grouping of a large number of cnidarian genes in a common clade (H) and the confirmation by chromosomal linkage that many of these genes are the results of tandem duplications in the *Nematostella* and *Acropora* genomes, indicate that these genes originate from a single ancestral gene. The presence of grouped cnidarian genes in the second long branch clade (R) together with representative of genes of all bilaterian clades suggest the presence of a second *Hes* gene, related to the *enhancer-of split* cluster genes of *Drosophila*. This clade is persistent when eliminating the sequences without Orange domain from the dataset but remains composed of genes evolving significantly faster than those of clade M. The grouping of genes of all bilaterian phyla in clade L, with the exception of a few presumably more derived annelid and echinoderm genes, is indicative of a single short-branch *Hes* in the bilaterian ancestor. A second clade of *enhancer-of-split* bilaterian genes is also present in both trees, supporting a putative second *Hes* gene. This clade is again composed of fast-evolving sequences, and comprises, surprisingly, ctenophore genes. It is therefore more questionable.

We conclude from the combined results of phylogenetic analyses and genomic organization that Hes superfamily is divided into five families, three of them being already present as a single gene in the urmetazoan (*Hes, Hey* and *Helt*). The evolution of the Hes family has been shaped by many independent lineage specific tandem duplication events. Is this situation often found in the gene family or is it highly unusual? We made parsimony reconstruction of the evolution of the character, number of gene copies, among more than 40 bHLH families (Additional file 3 B, C and D and data not shown). Those analyses revealed that the Hes family duplication rate is drastically superior compared to all other families. Thus, the presence of large numbers of *Hes* genes in a number of animal species represents a form of evolutionary convergence.

### *Platynereis* Hes superfamily members involved in two major, potentially ancestral, developmental processes: neurogenesis and segment addition

**Figure 6:**
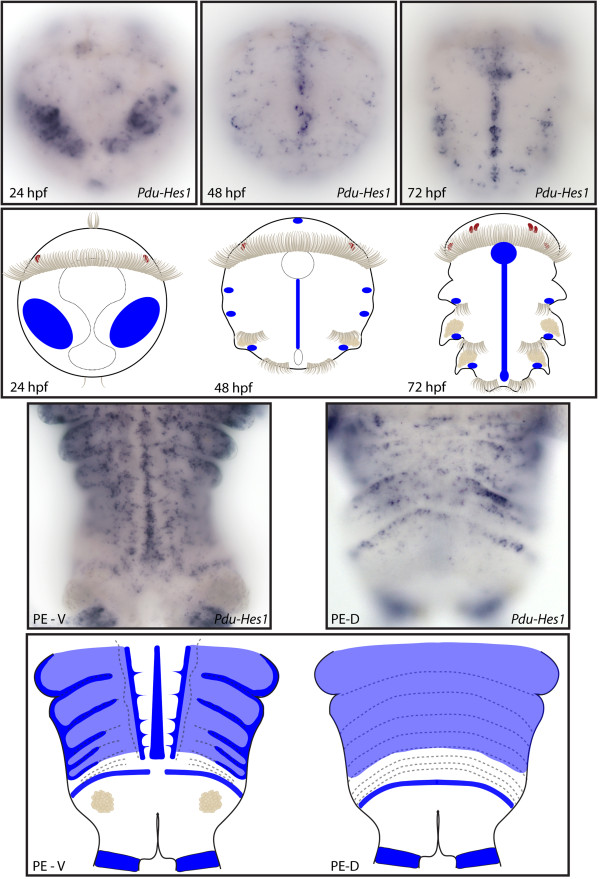
**Expression patterns of *Pdu-Hes1 *and their schematic representations in *Platynereis *larvae and during posterior elongation (PE).** Whole-mount *in situ* hybridizations (WMISH) for the different stages indicated on each panel are shown. *Pdu-Hes1* is first expressed in ectodermal columns at 24 h post fertilization (hpf) then restricted to the midline and segment epidermis lateral cells that form a more or less continuous line in each segment at 48 and 72 hpf. During PE, *Pdu-Hes1* is still expressed in the midline and the whole segment epidermis, (while more intensively at the segment boundaries and borders), but also in the ectodermal segment addition zone (SAZ) and at the basis of the anal cirri. In the dorsal part, its expression is in the form of broad stripes but some distance away from the SAZ, therefore in maturing segments. For the larvae, panels are ventral views (anterior is up). Expressions patterns during PE are shown for both sides (anterior is up), V = ventral, D = dorsal.

**Figure 7:**
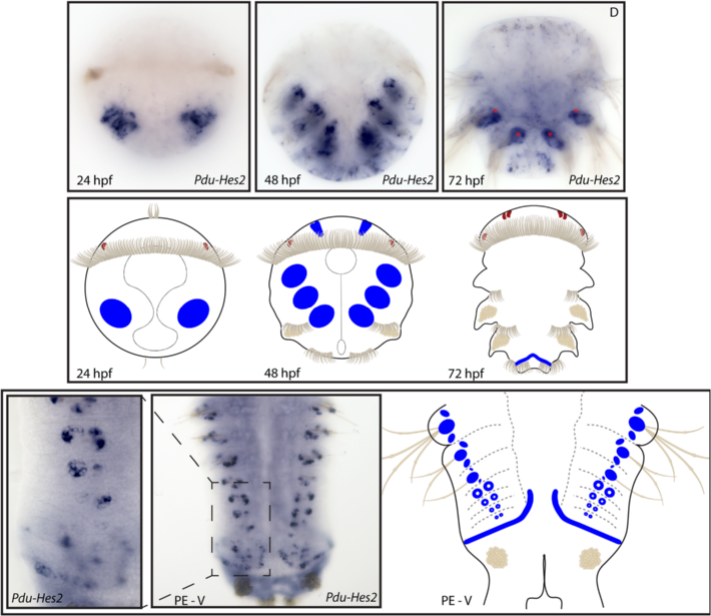
**Expression patterns of *Pdu-Hes2 *and their schematic representations in *Platynereis *larvae and during posterior elongation (PE).** Whole-mount *in situ* hybridization (WMISH) for the different stages indicated on each panel is shown. *Pdu-Hes2* is first expressed in two lateral ectodermal patches at 24 h post fertilization (hpf) and expands broadly in the 12 chaetal sacs, and in few cells of the brain at 48 hpf. At 72 hpf its expression is restricted to the dorsal segment addition zone (SAZ) located between the third segment and the pygidium (expressions in the silk glands, indicated by an asterisk, are an artifact). During PE, *Pdu-Hes2* is expressed very early, in the future chaetal sacs of each segment, in a ring-like shape, corresponding to the follicular cells of the follicles (that compose the chaetal sacs) with the deeper seating cells, the chaetoblasts, not stained. When chaetae protrude, the expression is in a larger spot at the basis of chaetae. Panels are ventral views (anterior is up) for the larvae and during PE.

**Figure 8:**
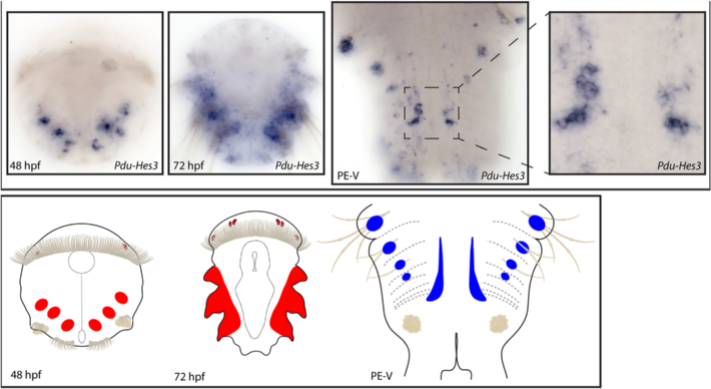
**Expression patterns of *Pdu-Hes3 *and their schematic representations in *Platynereis *larvae and during posterior elongation (PE).** Whole-mount *in situ* hybridization (WMISH) for the different stages indicated on each panel is shown. In the 48 and 72 h post-fertilization (hpf) trochophore larvae, *Pdu-Hes3* is expressed in the mesoderm of the (presumptive) parapodia. During PE, *Pdu-Hes3* expression covers the exterior borders of the ventral nerve cord (VNC), with a more large and intense expression in the VNC of the segments recently produced by the segment addition zone (SAZ). *Pdu-Hes3* is also expressed in ectodermal patches at the basis of the chaetae. Panels are ventral views (anterior is up) for the larvae and during PE.

**Figure 9:**
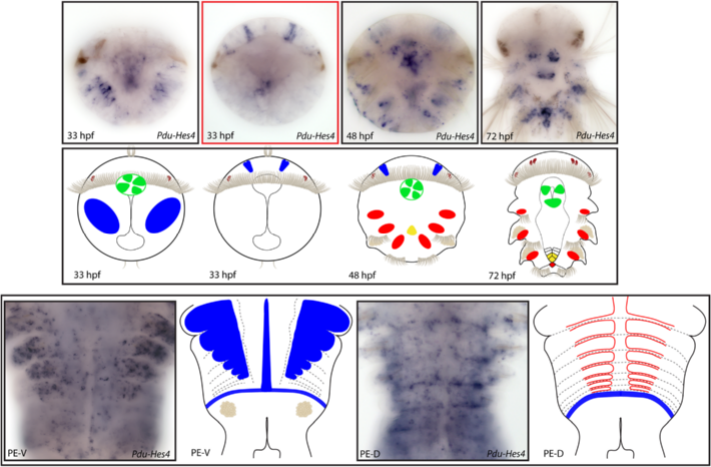
**Expression patterns of *Pdu-Hes4 *and their schematic representations in *Platynereis *larvae and during posterior elongation (PE).** Whole-mount *in situ* hybridization (WMISH) for the different stages indicated on each panel is shown. From 33 to 48 h post fertilization (hpf), *Pdu-Hes4* is expressed predominantly in two columnar brain cells in the dorsal part of the larvae, in the stomodeum and in the mesoderm of the (presumptive) parapodia. At 72 hpf, an intense expression is found in the developing cone-shaped midgut and mesodermal segment addition zone (SAZ). During PE, this gene is broadly expressed in the segment epidermis of well-developed segments (in the ventral side), in the midline, in the ectodermal SAZ as well as the dorsal blood vessels. For the larvae, panels are ventral views (anterior is up). Expressions patterns during PE are shown for both sides (anterior is up), V = ventral, D = dorsal. Deeper ventral view (different focus plane) is highlighted in red for 33hpf larvae.

**Figure 10:**
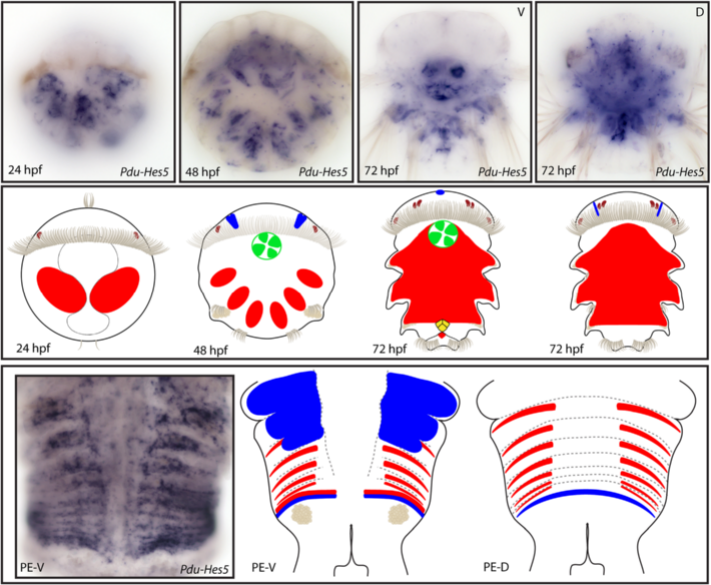
**Expression patterns of *Pdu-Hes5 *and their schematic representations in *Platynereis *larvae and during posterior elongation (PE).** Whole-mount *in situ* hybridization (WMISH) for the different stages indicated on each panel is shown. From 24 to 72 h post fertilization (hpf), *Pdu-Hes5* is expressed in mesodermal lateral blocks. It is first restricted to the future segments at 48hpf, and then extends to the back of the embryos and the mesodermal segment addition zone (SAZ) at 72 hpf. The brain, stomodeum and the developing cone-shaped midgut cells also expressed *Pdu-Hes5* respectively at 48 and 72 hpf. During PE, *Pdu-Hes5* expression is found in both the mesodermal and ectodermal SAZ, and extended to the segment epidermis and in mesodermal stripes at the anterior part of segments. For the larvae, panels are ventral views (anterior is up), excepted for a 72-hpf picture and schema. Expressions patterns during PE are shown for both sides (anterior is up), V = ventral, D = dorsal.

**Figure 11:**
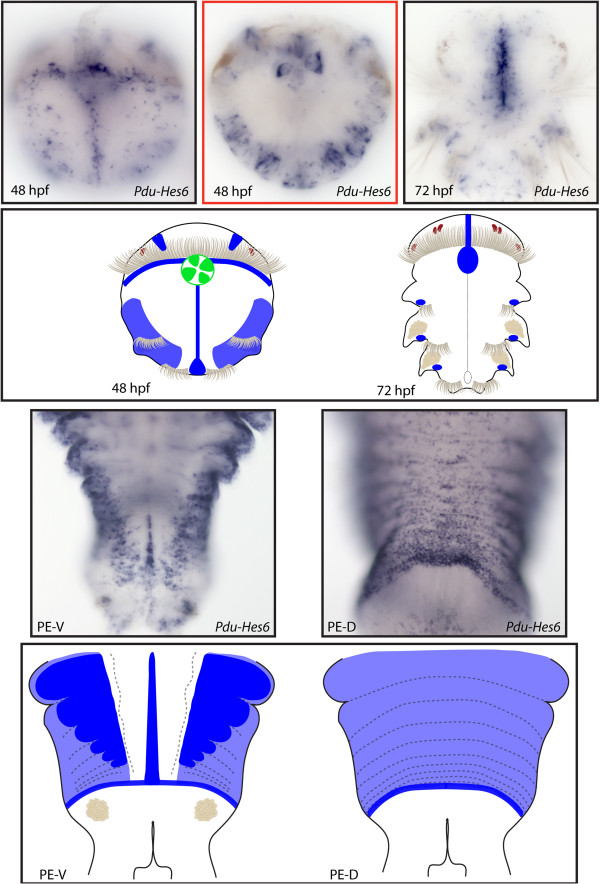
**Expression patterns of *Pdu-Hes6 *and their schematic representations in *Platynereis *larvae and during posterior elongation (PE).** Whole-mount *in situ* hybridization (WMISH) for the different stages indicated on each panel is shown. *Pdu-Hes6* is first expressed in ectodermal patches at 24 h post fertilization (hpf) then restricted to the midline, the stomodeum, two brain cells in the dorsal side and ectodermal lateral cells that form a more or less continuous line in each segment of the 48-hpf larvae. At 72 hpf its expression is greatly reduced and only concerns the midline plus minor patches in each segment. During PE, *Pdu-Hes6* is still expressed in the midline, but also in the ectodermal segment addition zone (SAZ) and in the whole segment epidermis. Expression in both the dorsal and ventral segment epidermis is in a striped fashion, with more intensive expression in the borders and boundaries of the segments. For the larvae, panels are ventral views (anterior is up). Expressions patterns during PE are shown for both sides (anterior is up), V = ventral, D = dorsal. Deeper ventral view (different focus plane) is highlighted in red for 48hpf larvae.

**Figure 12:**
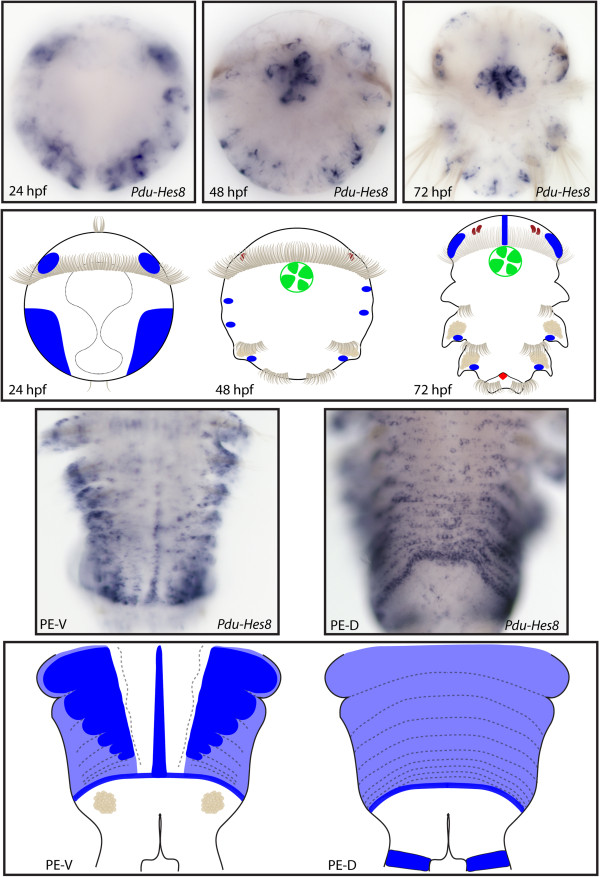
**Expression patterns of *Pdu-Hes8 *and their schematic representations in *Platynereis *larvae and during posterior elongation (PE).** Whole-mount *in situ* hybridization (WMISH) for the different stages indicated on each panel is shown. *Pdu-Hes8* expression is mainly restricted to the ectodermal cells, in lateral patches in the episphere and hyposphere of the 24hpf post fertilization (hpf) larvae. At 48 and 72 hpf, its expression is reduced in minor patches in each segment and in the stomodeum, plus brain cells and mesodermal segment addition zone (SAZ) cells at 72 hpf. During PE, *Pdu-Hes8* is expressed in the midline, and also in the whole segment epidermis, in the ectodermal SAZ and at the basis of the anal cirri. Expression in both the dorsal and ventral segment epidermis is in a striped fashion, with more intense expression in the borders and boundaries of the segments. For the larvae, panels are ventral views (anterior is up). Expressions patterns during PE are shown for both sides (anterior is up), V = ventral, D = dorsal.

**Figure 13:**
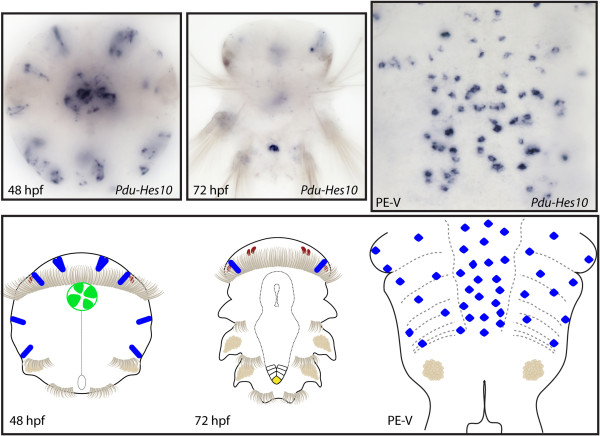
**Expression patterns of *Pdu-Hes10 *and their schematic representations in *Platynereis *larvae and during posterior elongation (PE).** Whole-mount *in situ* hybridization (WMISH) for the different stages indicated on each panel is shown. *Pdu-Hes10* is expressed in several brain cells from 33 to 72 h post fertilization (hpf), as well as in the stomodeum, disparate ectodermal cells (48 hpf) and gut cells (72 hpf). *Pdu-Hes10* has a salt and pepper expression pattern in the ventral nerve cord (VNC), plus an expression in disparate unknown cells of the forming parapodia, possibly corresponding to sensory or peripheral nervous system cells. Panels are ventral views (anterior is up) for the larvae and during PE.

**Figure 14:**
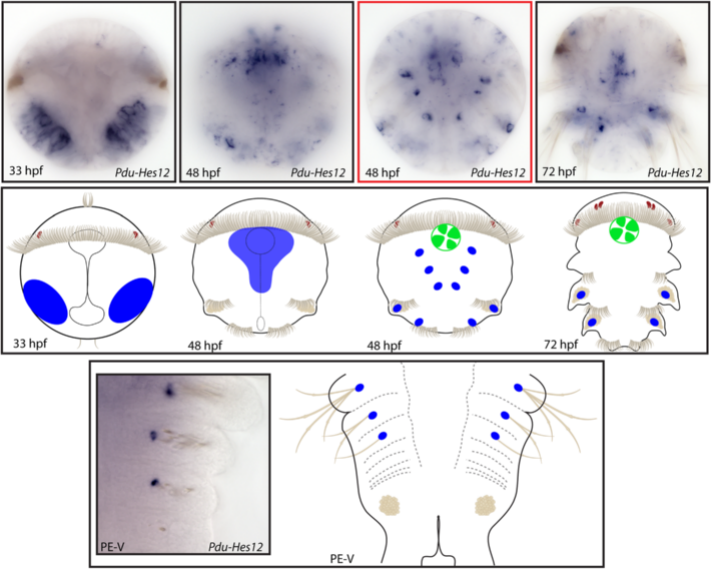
**Expression patterns of *Pdu-Hes12 *and their schematic representations in *Platynereis *larvae and during posterior elongation (PE).** Whole-mount *in situ* hybridization (WMISH) for the different stages indicated on each panel is shown. At 33 h post fertilization (hpf), *Pdu-Hes12* is expressed in two large lateral patches that become restricted in a weak expression in the ventral nerve cord (VNC) and around the stomodeum at 48 hpf. At this stage, as well as during PE, this gene is also expressed in a single cell at the bottom of each chaetal sac. An expression in the stomodeum is also evidenced at 48 and 72 hpf. Panels are ventral views (anterior is up) for the larvae and during PE. Deeper ventral view (different focus plane) is highlighted in red for 48-hpf larvae.

**Figure 15:**
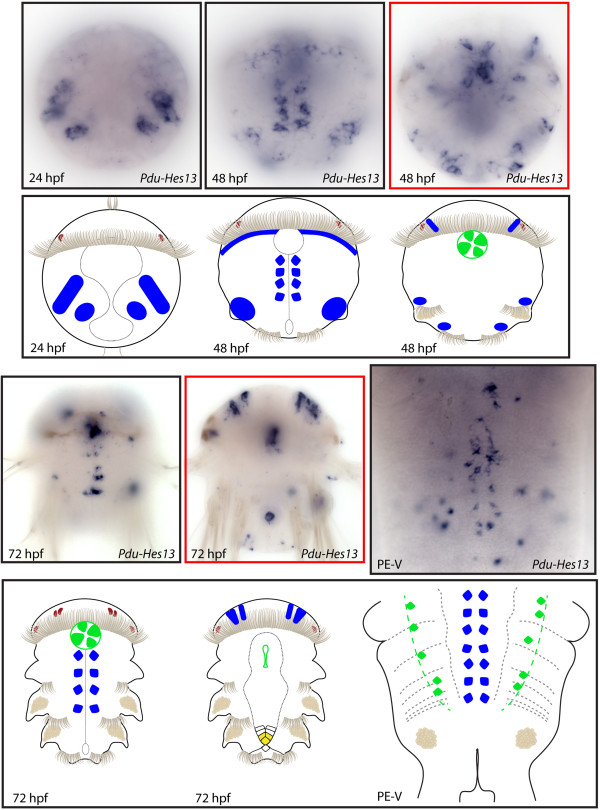
**Expression patterns of *Pdu-Hes13 *and their schematic representations in *Platynereis *larvae and during posterior elongation (PE).** Whole-mount *in situ* hybridization (WMISH) for the different stages indicated on each panel is shown. *Pdu-Hes13* is first expressed in four bilateral patches at 24 h post fertilization (hpf). At 48 hpf, *Pdu-Hes13* + cells of the ventral nerve cord (VNC), at the vicinity of the midline, are observed, in addition to few brain cells and stomodeum cells. At 72 hpf, the expression in cells around the midline, in the stomodeum and in the brain are maintained or extended (for the brain cells), and an additional expression in the cone-shaped midgut appears. During PE, this gene is still expressed in cells of the VNC around the midline plus in scattered gut cells. Panels are ventral views (anterior is up) for the larvae and during PE. Deeper ventral views (different focus plane) are highlighted in red for 48 and 72hpf larvae.

**Figure 16:**
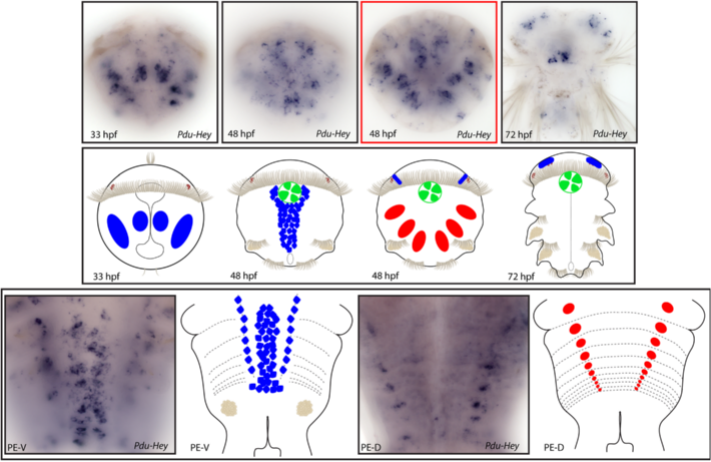
**Expression patterns of *Pdu-Hey *and their schematic representations in *Platynereis *larvae and during posterior elongation (PE).** WMISH for the different stages indicated on each panel is shown. *Pdu-Hey* is first expressed in four bilateral patches at 33 h post fertilization (hpf), two of them being in each side of the ventral midline. At 48 hpf, *Pdu-Hey* + cells are observed in a larger territory, surrounding the ventral nerve cord (VNC), in addition to few brain cells, stomodeum cells and mesodermal patches. At 72 hpf, the expression is maintained in brain and stomodeum cells. During PE, *Pdu-Hey* is expressed in the ganglions of the VNC of mature segments as well as in specific spots of the dorsal mesoderm. For the larvae, panels are ventral views (anterior is up). Expressions patterns during PE are shown for both sides (anterior is up), V = ventral, D = dorsal. Deeper ventral view (different focus plane) is highlighted in red for 48hpf larvae.

The unusual evolutionary history of the Hes family described above leads us to question when the multiple functions of *Hes* genes have been acquired during metazoan evolution and how these functions evolved. For that purpose, we monitored expression patterns of the lophotrochozoan *Platynereis Hes/Hey*-related genes at five different embryonic developmental stages (early, mid and late trochophore, metatrochophore and early nectochaete), and also during juvenile posterior elongation when new segments are added sequentially. The overall morphologies of the studied stages are shown in Additional file 4. Two of the analyzed genes: *Pdu-Hes7* and *Pdu-Hes9* show none or very weak and ubiquitous expressions at all studied stages (not shown). We investigated the presence of those two *Pdu-Hes* genes in six different transcriptomic databases (available with restricted access at http://4dx.embl.de/platy/). We found the presence of *Pdu-Hes9* exclusively in a 454 cDNA library obtained from juvenile heads. *Pdu-Hes9* appeared thus as an adult specific regulator, which is congruent with our non-conclusive *in situ* hybridization experiments on embryonic stages. *Pdu-Hes7* is found in two of the six transcriptomic databases and is thus presumably none or weakly expressed in the studied stages. Nevertheless, we cannot exclude the fact that technical limitations have prevented us from accessing a weak or very transient expression. These two genes will not be further described. *Pdu-Hes11* and *Pdu-Stich* expression patterns were obtained only at 72 hpf (Additional file 5). Expression patterns for relevant stages, as well as their schematic representations for *Pdu-Hes1*, *Pdu-Hes2*, *Pdu-Hes3*, *Pdu-Hes4*, *Pdu-Hes5*, *Pdu-Hes6*, *Pdu-Hes8*, *Pdu-Hes10*, *Pdu-Hes12*, *Pdu-Hes13* and *Pdu-Hey* are provided in Figures 6, 7, 8, 9, 10, 11, 12, 13, 14, 15, and 16 respectively. Detailed descriptions of those expression patterns are provided in the figure legends.

#### *Developmental expressions of* Platynereis Hes/Hey*-genes suggest an involvement in nervous system patterning*

All 13 *Hes/Hey*-related genes for which we obtained expression data for are expressed in cells that are crucial for the formation of the central and peripheral NS. These include widely distributed territories, such as the ventral midline, the ventral nerve cord (VNC), the PNS and some brain cells (Table 4). The midline corresponds to a specialized population of cells that demarcate the plane of bilateral symmetry between the two halves of the neurectoderm. This is the place where the edges of the proliferating trunk ectoderm meet and fuse during gastrulation [89]. We observed that *Pdu-Hes1, Pdu-Hes6, Pdu-Hes8* and *Pdu-Stich* are expressed there (Figures 6, 11, 12 and Additional file 5). Previous studies reported the specific expression of several genes in the larvae ventral midline cells such as *slit, sim* and *netrin*[90] but also a *wnt* gene (*Wnt 4*, [57]), two upstream regulators of the core PCP proteins (*fat* and *four-jointed*, [91]) and a microRNA (*mir92*, [92]). In *Drosophila*, *m3, m7, m*γ, and *m*δ (*E(spl)* genes) are specifically expressed in the embryonic midline and *m7* transcripts are present in the midline until the condensation of the nerve chord [93]. It has been shown that the ventral midline of protostomes is homologous to the floor plate (FP) of vertebrates, thus, in mouse, *Hes1* is present in the FP cells that are morphologically specialized cell populations at the ventral midline of the neural tube [94]. Consistent with the non-neurogenic property of FP cells, persistent expression of *Hes1*, which suppresses proneural gene expression, is required for the establishment of FP cell fate in mouse [95]. Functionally, the structure serves as an organizer sending a ventralizing signal to the neural tube, as well as to guide neuronal positioning and differentiation along the dorsoventral axis of the neural tube. Recently, it was proposed that *Platynereis* ventral midline may also act as an organizing center, which produces signals important for neuron production and the proper scaffolding of the VNC [91].

**Table 4:** **Summary of *Platynereis *** **expression patterns during embryonic and post-embryonic development**

		**Segmentation process**					**Nervous system patterning**				**Other organogenesis**				**Others**
	**Figure**	**SAZ**		**Stripes**		**Segment epidermis patterning**	**Midline**	**VNC**	**PNS**	**Brain cells**	**Chaetal sacs**	**Stomodeum**	**Midgut**	**Parapodia**	
	**Number**	**Ecto**	**Meso**	**Ecto**	**Meso**										
*Pdu-Hes 1*	6					x	x					x			24 hpf: ectodermal columns; 48 hpf: apical organ
*Pdu-Hes 1* PE		x				x	x								Anal cirri tentacles
*Pdu-Hes 2*	7	x								x	x				24 hpf: ectodermal patches
*Pdu-Hes 2* PE		x									x			x	Ectodermal columns
*Pdu-Hes 3*	8													x	
*Pdu-Hes 3* PE								x						x	
*Pdu-Hes 4*	9		x							x		x	x	x	
*Pdu-Hes 4* PE		x				x								x	Blood vessels
*Pdu-Hes 5*	10		x							x		x	x	x	
*Pdu-Hes 5* PE		x	x		x	x								x	
*Pdu-Hes 6*	11					x	x			x		x		x	
*Pdu-Hes 6* PE		x				x	x								
*Pdu-Hes 8*	12		x			x				x		x		x	
*Pdu-Hes 8* PE		x				x	x								Anal cirri tentacles
*Pdu-Hes 10*	13									x		x	x		
*Pdu-Hes 10* PE								x	x						
*Pdu-Hes 12*	14							x			x	x			
*Pdu-Hes 12* PE											x				
*Pdu-Hes 13*	15							x		x		x	x		24 hpf: ectodermal patches
*Pdu-Hes 13* PE								x							Dispersed gut cells
*Pdu-Hey*	16							x		x		x		x	
*Pdu-Hey* PE								x							

*Platynereis Hes/Hey*-related genes are mainly (but not only) expressed in cells, tissues, organs or structures that are related to the patterning of two major developmental processes: segmentation and nervous system patterning. Hpf, hours post fertilization.

In the early neurectoderm, five *Hes/Hey*-related genes (*Pdu-Hes3*, *Pdu-Hes10*, *Pdu-Hes12*, *Pdu-Hes1* and *Pdu-Hey*) are active in neurogenic cells distinct from the midline cells (Figures 8, 13, 14, 15 and 16)*.* Among them, *Pdu-Hes3* and *Pdu-Hes10* are exclusively expressed in the VNC during PE (Figures 8 and 13) in opposition to *Pdu-Hes12* only found in the neurectoderm at 48 hpf (Figure 14). *Pdu-Hes1* is active in both the early and late neurectoderm with an expression in bilateral columns, in addition to the midline, as well as in intersegmental stripes in the prospective VNC only, that persists as VNC ganglia differentiate (Figure 6). During PE, we observed that expressions of both *Pdu-Hes13* and *Pdu-Hey*, are maintained in the ganglions of the VNC of maturing segments, suggesting that those genes, also involved in later neurogenesis, mark differentiated neurons or neurons in the course of differentiation. As these expressions are highly restricted compared to the *Elav* pattern [90], a marker of the whole VNC, we suggest that those genes are expressed in a sub-population of neurons or neurons in differentiation of the VNC.

In the brain, 10 *Hes/Hey*-related genes are active in rather small specific subsets of cells. With the term brain, we refer here to the cells that occupy the dorsal half of the episphere of *Platynereis* larvae [96] and take part in the formation of the worm prostomium. Most *Pdu-Hes* are expressed in pairs of columnar cells in the dorsal part of the episphere of the larvae (Figures 9, 10, 11, 12, 13, 15, 16, and Additional file 5). Most of these expressions in pairs of columnar cells look similar, but we cannot establish whether the same cells expressed several *Hes* genes. As the precise characterization of the cells that express those genes is not the main focus of our study, we did not detail further these expressions. We nevertheless noticed that such precise *Hes/Hey*-related expressions in brain are also observed in several other organisms, such as *deadpan* in the *drosophila*[97]. Similarly, in zebrafish, several *Hes*-related genes (*Her3, Her5, Her11*[12]) are known to be expressed in the brain, specifically in the midbrain-hindbrain boundaries they contributed to form. Above those expressions in the central nervous system ((CNS): VNC and brain cells), one *Hes* (*Pdu-Hes10)* is also expressed in disparate unknown cells that also do not harbor a precise bilateral pattern and that could possibly be precursors of sensory cells of the PNS (sensory organs and neurons) (Figure 13).

One of the striking features of this study is that all *Hes/Hey*-related genes from which we obtained expressions are found in one or more cells/tissues/structures related to the NS patterning. Nevertheless, their expressions are often specific to a category of cells. Indeed, few genes found in the VNC or PNS are also expressed in brain cells. Notably, the *Hes/Hey*-related genes expressed in the ventral midline cells are almost never expressed in the VNC or PNS. This last point is in accordance to previous results showing that midline markers are never expressed in neurectoderm, suggesting a non-neurogenic property of *Platynereis* midline cells, similar to the FP cells. Those results may also suggest the involvement of a combinatorial code of *Hes/Hey*-related genes in the *Platynereis* NS patterning. In the annelid *Capitella teleta*, three *Hes* genes (of the six we identified, Table 3) have been previously studied and two of them (*CapI-Hes2* and *CapI-Hes3*) are localized in a small part of the developing brain in larvae and in the forming ganglia of the VNC of juveniles [47]. *CapI-Delta* and *CapI-Notch* expression patterns in the larvae and juvenile are reminiscent of *CapI-Hes2* and *CapI-Hes3* ones, except for *CapI-Delta*, absent in the VNC of the juvenile. Due to the pivotal role of *Hes/Hey*-related genes in vertebrate and arthropod neural development in regulating proliferation, differentiation and specification of neural stem cells in a Notch-dependent manner, results observed in *Capitella* may reinforce the view of an ancestral function of the Notch signaling pathway in patterning the NS of bilaterians. But the situation is maybe not so simple, as in *Platynereis*, neither *Notch* nor *Delta* seems to be expressed in cells, tissues or structures related to the NS (Gazave and Balavoine, Notch signaling in the annelid *Platynereis*, in preparation). Thus the 13 *Hes/Hey*-related genes of *Platynereis* that appears to be involved in neurogenesis may act in a Notch-independent manner and the nature of their regulators is still a totally open question.

#### Platynereis *genes expression patterns support an implication in segmentation processes*

Among the 13 *Hes/Hey*-related genes from which we obtained precise expression data, seven are localized in structures (SAZ), patterns (expression in stripes) or territories (segment epidermis) that suggest an involvement in the process of segment formation (Table 4, Additional file 5). The segmentation process can be virtually divided into three major steps in both arthropods and vertebrates and presumably in annelids: the axis growth, the specification of a segmental periodicity and the axial patterning of individual segments. In *Platynereis*, the first step, the production of an elongated anterior-posterior axis, relies on the presence of both ectodermal and mesodermal stem cells, called teloblasts, in a specific ring-shaped posterior zone: the SAZ [82]. During posterior elongation, six *Hes* genes (*Pdu-Hes1, 2, 4, 5, 6* and *8*) are expressed in a 1- to 3/4-cell-wide ring of ectodermal cells, immediately anterior to the pygidium, in the SAZ (Figures 6, 7, 9, 10, 11 and 12). The ring, most clearly visible on the dorsal side of the worms, extends to their ventral face but is in most cases interrupted in the ventral-most part of the ectoderm, as illustrated by the pattern of *Pdu-Hes2* (Figure 7)*.* This ring-like expression is similar to what is observed for several genes already described in ectodermal teloblast-like cells [65,82]. *Pdu-Hes5* is not restricted to this ring-like ectodermal expression but is also expressed in a ring-like group of posterior mesodermal cells located immediately anterior to the pygidium boundary, in the ventral side (Figure 10). Several genes that are expressed in the ectodermal SAZ during PE, are also found in a group of internal cells located at the border between the 3^rd^ chaetigerous segment and the forming pygidium at 72 hpf (*Pdu-Hes4, 5, 8* and *11;* Figures 9, 10, 12 and Additional file 5), or in a ring-like fashion, on the dorsal ectoderm only (Figure 7)*.* These internal cells, first reported by Rebscher *et al*. [98,99] as forming a prospective mesodermal posterior growth zone are thought to be derived from the primary mesoblasts of the 4d lineage. Gazave *et al*. [82] provided expression data for these cells, for more than 20 genes (mainly RNA-binding proteins and transcription factors) involved in the formation, behavior, or maintenance of stem cells in other metazoan organisms. These cells are proliferative, correspond most probably to the mesodermal component of the prospective SAZ and at least a part of it comprises stem cells [82]. Thus, those *Pdu-Hes* expressions are very similar to what we have reported before, both at 72 hpf and during PE, suggesting that they are expressed in posterior ecto and/or meso teloblast-like stem cells of the SAZ, involved in the PE process. Although PE also occurs in some groups of non-segmented animals, this process has been so far mainly described in segmented bilaterian animals, many of which form most of their body axis through the sequential posterior addition of segments [100].

Interestingly, for one gene, *Pdu-Hes5,* expression is not only restricted to the ecto and mesodermal cells of the SAZ, but continued in mesodermal stripes, well before any trace of segmentation is visible (Figure 10). These stripes are persistent in differentiating segments and positioned in the anterior part of each segment. These mesodermal stripes also extend in the lateral sides of the trunk but are interrupted ventrally and dorsally at the level of the unsegmented ventral and dorsal vessels, respectively. A previous study of *Platynereis* reported the expression of mesodermal stripes for four genes of the NK family (*Pdu-Msx, Pdu-Lbx, Pdu-Tlx* and *Pdu-NK1*) that have been proposed to be associated with the segmented mesodermal epithelia that surround the coelomic cavities [101]. One of them, *Pdu-NK1* is precisely located at the anterior part of the segment, like *Pdu-Hes5*. The expressions of those four genes are complementary, covering most of the mesodermal segments, suggesting that they might be working in a concerted way to pattern the A/P polarity of individual mesodermal segments. A similar role for *Pdu-Hes5*, whereas no others *Hes* are expressed in mesodermal stripes, could be thus also proposed.

Five *Hes/Hey*-related genes (*Pdu-Hes1, 4, 5, 6,* and *8*) are also expressed in various patterns in the segment epidermis of the larvae and/or during PE. These genes are not expressed in stripes in the vicinity of the SAZ, but in maturing segments suggesting that they are not early players in the segmental patterning (Figures 6, 9, 10, 11, 12). These late expressions are probably indicative of a role in segment differentiation rather than in segment early patterning.

In this study, we show that seven *Hes/Hey*-related genes are expressed in structures related to the segmentation process. Although five of them are found in two categories of expression patterns (that is, SAZ and segment epidermis), only one gene, *Pdu-Hes5,* is expressed in both the ectodermal and mesodermal SAZ but also in mesodermal stripes and in the segment epidermis. This leads us to propose that *Pdu-Hes5* may be a key element acting during the axial patterning of *Platynereis* segments. The six other *Hes/Hey*-related genes expressed in structures related to the segmentation are localized in the teloblasts of the SAZ. The mitotic behavior of *Platynereis* teloblasts is coordinated to a certain degree [82] and they presumably undergo asymmetric divisions as leech teloblasts do [102]. One can suppose that those genes may be involved in such cellular processes and so have a role in the specification of a segmental periodicity. This would be reminiscent of the vertebrate situation, in which *Hes/Her* genes are periodically expressed in a wave-like fashion in the presomitic mesoderm (PSM) and are the main elements of the molecular clock that, through the control of Notch, induce somite formation [24-26]. A possible involvement of the Notch/Delta pathway in the segmentation of an annelid has been previously questioned [47]. During *Capitella teleta* larval development, neither *CapI-Hes1/2/3*, nor *CapI-Delta* and *CapI-Notch* are expressed in a striped pattern, suggesting they are not involved in the formation of the larval segments. Nevertheless, all of them are expressed in the mesodermal SAZ during PE of the juvenile, a fact that can be interpreted in favor of a role of these genes in the formation of segments during PE. In *Platynereis*, expression pattern during PE of *Notch* and *Delta* are in agreement with such a hypothesis (Gazave and Balavoine, Notch signaling in the annelid *Platynereis*, in preparation). If *Pdu-Hes5* is a, direct or not, target gene regulated by the Notch pathway in this segmentation process is an issue not yet resolved.

#### *All expressed* Hes/Hey*-related genes in* Platynereis *are involved in diverse organogenesis processes in addition to segmentation and nervous system patterning*

All 13 *Hes/Hey*-related genes from which we obtained expression data are expressed in specific organs or structures, such as chaetal sacs, stomodeum, midgut and parapodes (Table 4)*.* Thus, *Pdu-Hes4, 5, 10, 13* and *Pdu-Stich* are expressed in the developing cone-shaped midgut at 72 hpf (Figures 9, 10, 13 and 15, Additional file 5), while for *Pdu-Hes10* and *13*, this expression is restricted to specific round cells. Ten *Hes/Hey*-related genes (*Pdu-Hes1, 4, 5, 6, 8, 10, 11, 12, 13* and *Pdu-Hey*) are also expressed in the stomodeum of the larvae. Numerous *Hes/Hey*-related genes are found to be expressed more or less broadly in the parapodia, of the 72 hpf larvae and also during PE, in very different ways from one gene to another (Figures 7, 8, 9, 10 and 16).

Two *Hes/Hey*-related genes have an intriguing localization. These are *Pdu-Hes2* and *12* that are very specifically and intensively expressed in presumptive chaetal sac anlagen (Figure 7 and 14). Chaetae are chitinous bristle-like structures displayed by the annelid parapodes. At 48 hpf, chaetae do not protrude from the epidermal layer but grow internally in the chaetae sacs. There are 12 chaetal sacs in the trochophore, two per hemi-segment, located laterally. In a lateral view, each pair of ventral and dorsal sacs corresponds to the chaetal sac of the future neuropode and notopode of the parapodia. While *Pdu-Hes2* and *12* are both expressed in the same areas corresponding to the chaetal sac, their patterns are different. Indeed, *Pdu-Hes2* is expressed in 12 larges patches corresponding to a large proportion of the chaetal sac cells (Figure 7). In contrast, *Pdu-Hes12* expression is restricted to 12 spots of very few cells, presumably just one (6 groups in the ventral part and 6 in the dorsal one) (Figure 14) that sit at the internal tip of the chaetal sac. Morphological and ultrastructural studies revealed that chaetae emerge from epidermal follicles that in turn form the chaetal sacs. Furthermore, each follicle consists of one basal chaetoblast and several laterally surrounding follicle cells [103]. As a consequence, it appears that *Pdu-Hes2* and *12* are expressed differentially in the different cell-types of the follicles forming the chaetal sacs, *Pdu-Hes12* being found in a unique cell in each chaetal sac while *Pdu-Hes2* is located in the surrounding follicle cells. During PE, the situation seems similar, with precise expression at the basis of the chaetal sac harboring the already emerged chaetae, for *Pdu-Hes12. Pdu-Hes2* is expressed very early during PE, long before the protrusion of the chaetae, in the recently produced segment.

Expression of annelid *Hes* in the chaetal sac was previously reported in *Capitella teleta*[47]. Indeed, *CapI-Hes2* expression coincides with those of *CapI-Delta* and *CapI-Notch* in the presumptive chaetal sacs. Those expression patterns appear just after the segments form, and their detection ceases prior to the appearance of chaetae and suggest a role (direct or not) of the Notch pathway in chaetogenesis. In *Platynereis*, *Delta* and *Notch* are also expressed in the chaetal sacs (Gazave and Balavoine, Notch signaling in the annelid *Platynereis*, in preparation) supporting the idea that the involvement of Notch signaling in chaetal development may be an ancestral feature, at least, for annelids.

## Conclusions

### Gene duplication in the Metazoan Hes superfamily: insights from the *Platynereis* expression data

Gene duplication is one of the major mechanisms for the origin of functions of new genes and it is now well-known that the refashioning of duplicated genes is a great contributor to the origin of the evolutionary novelties [61]. Taking into account both evolutionary history of the family and expression data from *Platynereis*, we propose here two hypotheses to explain how gene duplication occurred in the Hes superfamily in metazoan.

One possibility to explain how this family is so prolific compared to other bHLH family (Additional file 3) is a high frequency of gene duplication events specifically for the Hes superfamily - a sort of hotspot of duplication, that could be explained by the presence of repeated sequences or late-replicating regions in the genomic area of *Hes* genes, which will raise the recombination rate, compared to other gene families [104]. Among the very high number of copies generated by the multiple duplications, a minority of them will become fixed by selection [58]. Nevertheless, such a hotspot process should produce large numbers of gene copies that become pseudogenes. In the species studied in this analysis we failed to find any evidence of pseudogeneization, such as in frame stop codons. Therefore, we cannot conclude that the high number of gene copies in some lineages lies in a high frequency of duplications rather than in a high retention rate of duplicated genes.

As a functionally indistinguishable duplicate has no chance to be fixed, two main models have been proposed to explain such a counterintuitive state: the neofunctionalization model and the DDC process [58]. The neofunctionalization model proposes that the accumulation of neutral mutations in both copies of the duplicated gene will rapidly cause the appearance of a new function in at least one of them, and thus a favorable selective context to retain both copies. However, it seems unlikely that such a process could by itself explain the multiplicity of *Hes* gene copies in some lineages, because this would also imply the repeated appearance of similar functions in different lineages. The DDC model relies instead on the presence of an ancestral gene that carried out pleiotropic roles. At first glance, *Platynereis Hes* genes are expressed in a variety of cells and territories. A comprehensive overview however suggests that they are mainly involved in three main processes of annelid development: segmentation, neuron subtype-differentiations and chaetogenesis. This is congruent with the presence of an ancestral *Hes* gene carrying a multiplicity of functions. We also observed three cases of combinatorial patterns of *Hes* genes among the *Platynereis* development. The first one is revealed by the multiples *Hes* genes that may be involved in the NS patterning, the *Pdu-Hes* expressed in the midline cells are never expressed concomitantly with others *Hes* genes found in the VNC, PNS or brain cells. Also noticeable is the combination of two *Hes* genes (*Pdu-Hes2* plus *Pdu-Hes12*) in the chaetal follicles. The addition of *Pdu-Hes2+* and *Pdu-Hes12+* cells exactly corresponds to most cells of the chaetal follicles. In a similar way, *Pdu-Hes6* and *Pdu-Hes8* have comparable expression patterns, but not strictly identical, suggesting also a duplication event but more recent. These three specific cases are arguments in favor of the DDC model during Hes superfamily evolution. A similar situation has been previously proposed for the *Branchiostoma Hairy* clustered genes [53].

**Figure 17:**
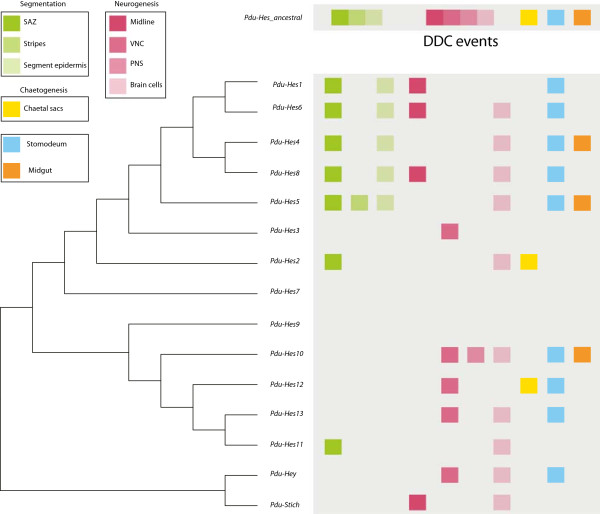
**Scenario of *Platynereis Hes/Hey*-related genes subfunctionalisation after duplications from a multifunctional *Pdu-Hes*-ancestral gene.** Phylogenetic relationships of *Platynereis Hes/Hey*-related genes are represented in a simple tree in front of which expression patterns are mapped. Expression patterns related to segmentation process, neurogenesis, chaetogenesis, in the stomodeum and the midgut are respectively coded in green, pink, yellow, blue and orange. A putative ancestral multifunctional *Platynereis Hes*-like gene is represented. Present expression patterns and evolutionary history of *Hes/Hey*-related genes may be the result of numerous duplication-degeneration-complementation (DDC) events.

Following this DDC hypothesis, a plausible schematic representation of expression territories in the multifunction ancestral *Pdu-Hes* is proposed in the Figure 17, as well as representation of specific patterns for each *Platynereis Hes/Hey*-related gene. When positioning *Platynereis Hes* expression in front of a tree of *Platynereis Hes* phylogenetic relationships, an intriguing pattern is revealed. Indeed, all but one genes expressed in territories related to the segmentation process are grouped together in a clade constituting the less divergent *Hes* (see phylogenetic part of the results and discussion). This clade also included all but one gene that harbor an expression in the midline. The more divergent *Hes*, which mainly correspond to those that have lost the Orange domain in the course of evolution are grouped together and are involved mainly in the NS patterning. Notably, the two markers of chaetogenesis are found in both clades.

Integrating both phylogenetic and expression data led us to hypothesize that an ancestral multifunctional *Pdu-Hes* gene has undergone duplication-degeneration-complementation processes, each gene copy ensuring their maintenance in the genome by subfunctionalisation. Nevertheless, we cannot totally exclude the possibility of a certain amount of neofunctionalisation and therefore a combination of those two genetic mechanisms to shape a complex evolutionary history as highlighted by the Hes superfamily case.

### A still open question: are the Hes superfamily members ancestrally regulated by Notch?

Can the *Hes/Hey*-related genes be considered canonical target genes of the Notch pathway? As highlighted in the Introduction, most studies of the *Hes/Hey*-related genes have been performed in the context of the analysis of the Notch signaling pathway. This biased point of view has led many authors to consider as a general rule their regulation by the Notch pathway, characterizing the *Hes/Hey*-related genes as canonical targets of the pathway. However, it should be stressed that the HES proteins, in contrast to Su(H) are not core components of the Notch pathway, and are used as transcriptional outputs of the pathway in some but not all Notch-dependent processes. Also few Notch-independent expressions and functions have been reported and recent studies have demonstrated that crosstalk between Notch and other major signaling pathways, such as fibroblast growth factor (FGF), bone morphogenetic protein (BMP) and transforming growth factor (TGF)-β, results in the regulation of some *Hes* or *Hey* genes in a Notch-independent fashion [10,105]. Thus a systematic regulation of *Hes/Hey*-related genes by Notch should not be expected while *Hes* Notch-independent roles have been marginally explored so far. As shown in this study, the last common ancestor of metazoan possessed at least three *Hes/Hey*-related genes and the Hes family underwent a large expansion in the course of the evolution of each lineage, resulting in the presence of numerous *Hes* paralogues in the present species. Unfortunately, expression and/or functional data are only known for a very small proportion of them, preventing a detailed picture of the *Hes/Hey*-related genes regulation versus non-regulation by Notch at a metazoan scale. We also know that the Notch pathway is a metazoan innovation, presumably already functional in the last common ancestor (LCA) [18]. We propose that a regulation by Notch of *Hes/Hey*-related genes was already present at the dawn of metazoan diversity but does not imply that all *Hes/Hey*-related genes that frequently appeared during metazoan evolutionary history retained this regulation. Studying all the *Hes/Hey*-related genes repertory of a species, as it was performed for *Branchiostoma*[53] and *Platynereis* (this study, Gazave and Balavoine, Notch signaling in the annelid *Platynereis*, in preparation ) will help to obtain a more realistic picture of *Hes/Hey*-related genes and Notch relationships.

## Abbreviations

aLRT: approximate likelihood ratio test; bHLH: basic helix-loop-helix; CSL: CBF1, Su(H): Lag-1; CNS: central nervous system; DDC: duplication-degeneration-complementation; Espl: Enhancer of split; EST: expressed sequence tag; FP: floor plate; HDAC: histone deacetylase; Hes: Hairy/enhancer of Split; Hey: Hairy/Enhancer of Split related with YRPW motif; hpf: hours post fertilization; Mitf: microphthalmia-associated transcription factor; ML: maximum likelihood; NICD: Notch intracellular domain; NS: nervous system; PE: posterior elongation; PNS: peripheral nervous system; SAZ: segment addition zone; SREBP: Sterol regulatory element binding protein; Su(H): Suppressor of Hairless; TF: transcription factor; VNC: ventral nerve cord; WMISH: whole-mount *in situ* hybridization; PBS: phosphate-buffered saline.

## Competing interests

The authors declare that they have no competing interests.

## Authors' contributions

EG carried out most of the molecular experiments (gene cloning, *in situ* hybridizations and imaging). AG performed part of the gene cloning. EG and GB jointly performed genome research, genomic analyses and phylogenetic analyses. EG and GB conceived and designed the study, analyzed data, wrote the first draft of the manuscript and were involved in editing the final version of the manuscript. All authors read and approved the final manuscript.

## Supplementary Material

Additional file 1: Table S1 Detailed information about the sequences of *Hes/Hey*-related genes used in our study. For each sequence, the species and lineage to which it belongs, the sequence name, the presence or absence of the main domains (basic helix-loop-helix (bHLH) and Orange) as well as the genomic localization (when available) are provided. NR = non relevant, no genomic localization data are available. Stars indicate the presence of clustered genes located in close genomic localization, with four genes in the case of Lgi168394c and five genes in the case of DreHer4c. Click here for file

Additional file 2: Table S2 Information about the physical linkages between *Hes/Hey*-related genes in 14 species among metazoans. In this table, we report the different *Hes/Hey*-related genes that are physically linked for each species, the genomic scaffolds (or chromosome) to which they belong, their position in these scaffolds as well as their strand. Click here for file

Additional file 3: Figure S1 Parsimony reconstruction analysis of character evolution based on a consensus Metazoan phylogenetic tree. The characters used in this analysis are the numbers of genes per basic helix-loop-helix (bHLH) family per species. Each character state is mentioned by a color code. Double-colored branches indicate non-determination of character state in the branch. The squares below taxon names give character state in the considered taxon; no square means unknown/missing data (in this case, character-state in the corresponding branch is optimized according to character-states in related taxa). A = Hes family: 1 to 22 *Hes* genes are present in the sampling dataset, the *Urmetazoan* presumably possessed one *Hes* gene and many duplications occurred. Gene loss is evidenced in one case. B = Hey family: 0 to 3 *Hey* genes are present in the sampling dataset, the *Urmetazoan* presumably possessed one *Hey* gene and 1 to 2 duplications occurred in the lineage leading to *Capitella teleta* and (*Danio Rerio + Homo sapiens*) clade only. Gene losses are evidenced in three cases. C = NeuroD family: 0 to 4 *NeuroD* genes are present in the sampling dataset, *Urbilateria* presumably possessed one *NeuroD* gene and duplications occurred in the lineage leading to (*Danio rerio + Homo sapiens*) clade. Gene loss is evidenced in one case. D = Clock family: 0 to 3 *Clock* genes are present in the sampling dataset, the *Urmetazoan* presumably possessed one *Clock* gene and one to two duplications occurred in several lineages. Gene loss is evidenced in one case. Click here for file

Additional file 4: Figure S2 Schematic drawings of *Platynereis dumerilii* general anatomy. Larval developmental stages studied as well as post-caudal regeneration posterior elongation process are shown. Those drawings are used in the main figures of the article for an easier comprehension of the expression patterns. A = 24 h post fertilization (hpf), ventral view; B = 33 hpf, ventral view, C = 48 hpf, ventral view; D = 72 hpf, ventral view (focusing on the neurectoderm); D = 72 hpf, deeper ventral view (focusing on internal structures such as the SAZ); E = post-caudal regeneration posterior elongation process, dorsal view; E’ = post-caudal regeneration posterior elongation process, ventral view. Ac = anal cirri; Ae = adult eye; At = apical tuft; bla = blastopore; ch = chaetae; Le = larval eye; Mg = midgut; Mid = midline; Para = parapodia; Pt = prototroch; Py = pygidium; S1 = 1^st^ segment; S2 = 2^nd^ segment; S3 = 3^rd^ segment; S = stomodeum; SAZ = segment addition zone; Telo = telotroch; VNC = ventral nerve cord. Click here for file

Additional file 5: Figure S3 Expression patterns of *Pdu-Hes11* and *Pdu-Stich* at 72 h post fertilization (hpf). Whole-mount *in situ* hybridization (WMISH) for the 72hpf stage is shown. *Pdu-Hes11* is expressed in various brain cells, stomodeum cells and mesodermal patches. In addition *Pdu-Hes11*+ cells are also observed in the segment addition zone (SAZ). *Pdu-Stich* is expressed in the midline cells, in various brain cells and mesodermal patches. Panels are mostly ventral views (anterior is up). A dorsal view (D) is also shown for *Pdu-Stich.* Click here for file
