# Supplementary figures and images for: History of a prolific family: the Hes/Hey-related genes of the annelid Platynereis
Source: EvoDevo. 2014 Sep 5;5:29. doi: 10.1186/2041-9139-5-29 (PMC4172395; doi:10.1186/2041-9139-5-29)

A) HES

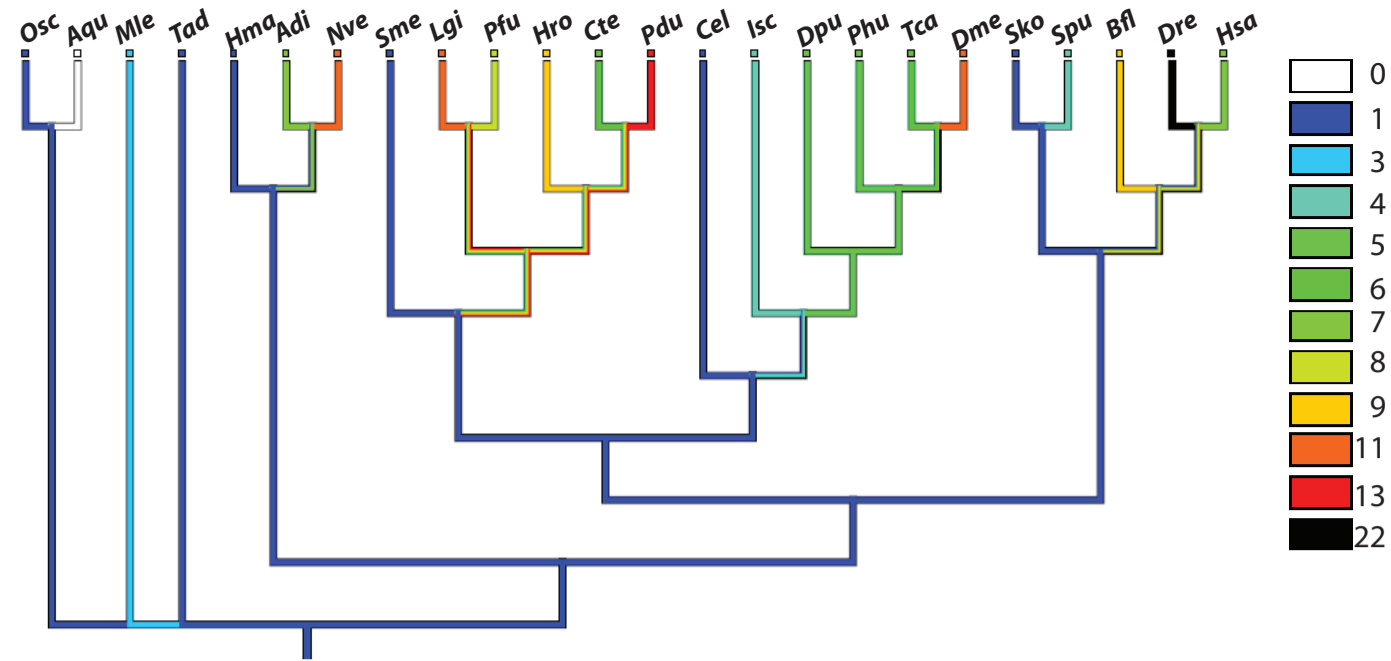

B) HEY

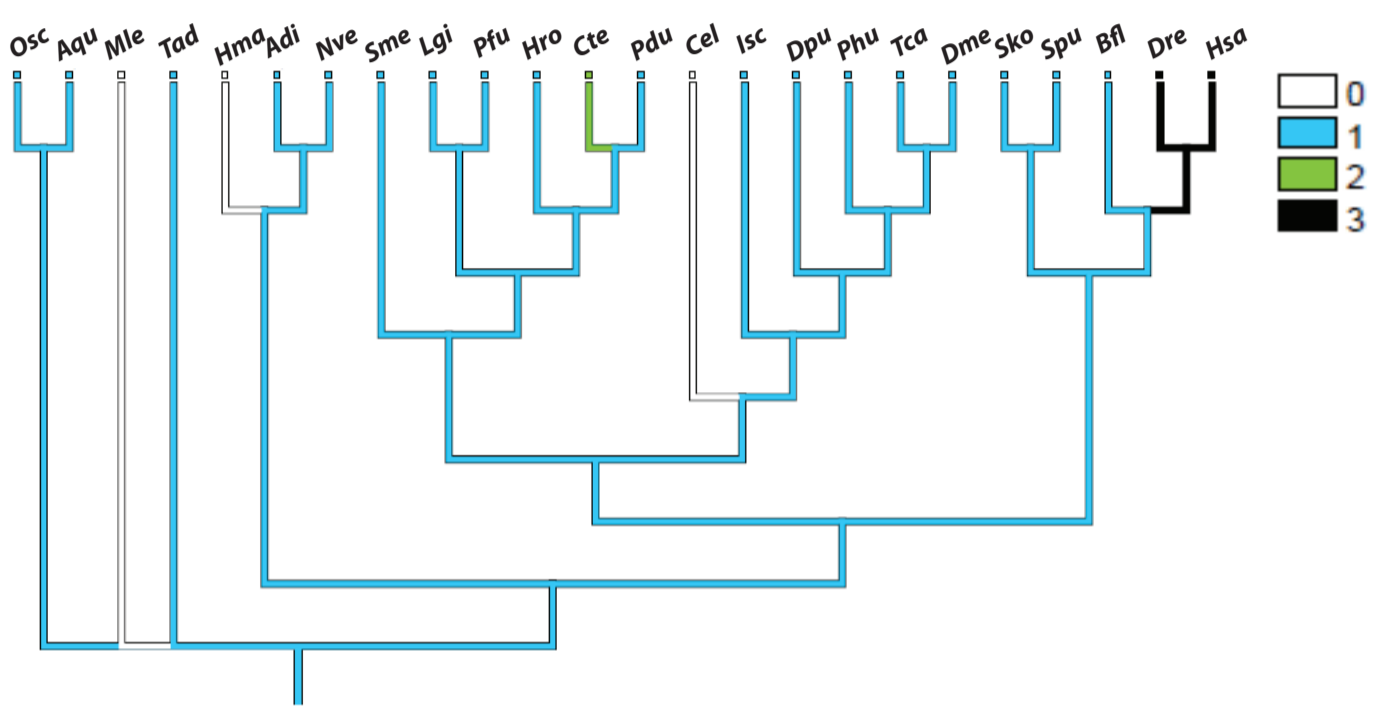

C) NeuroD

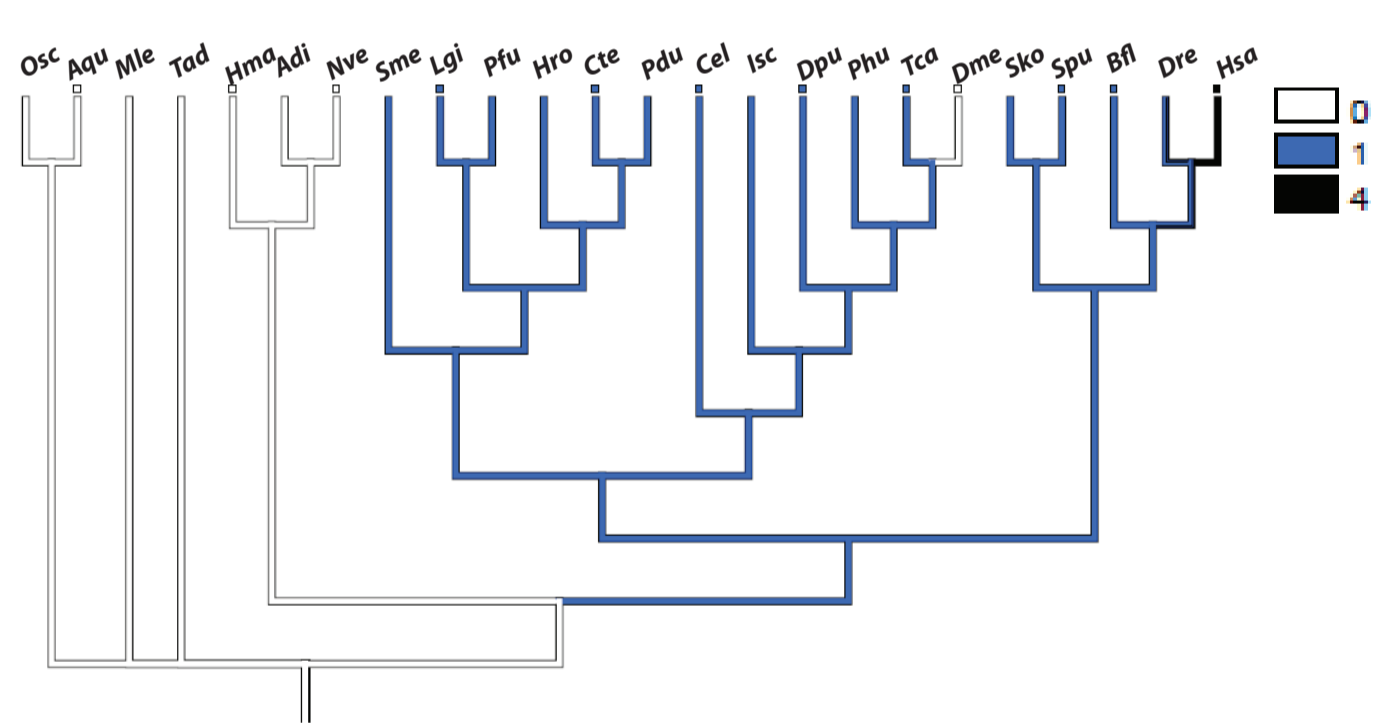

D) Clock

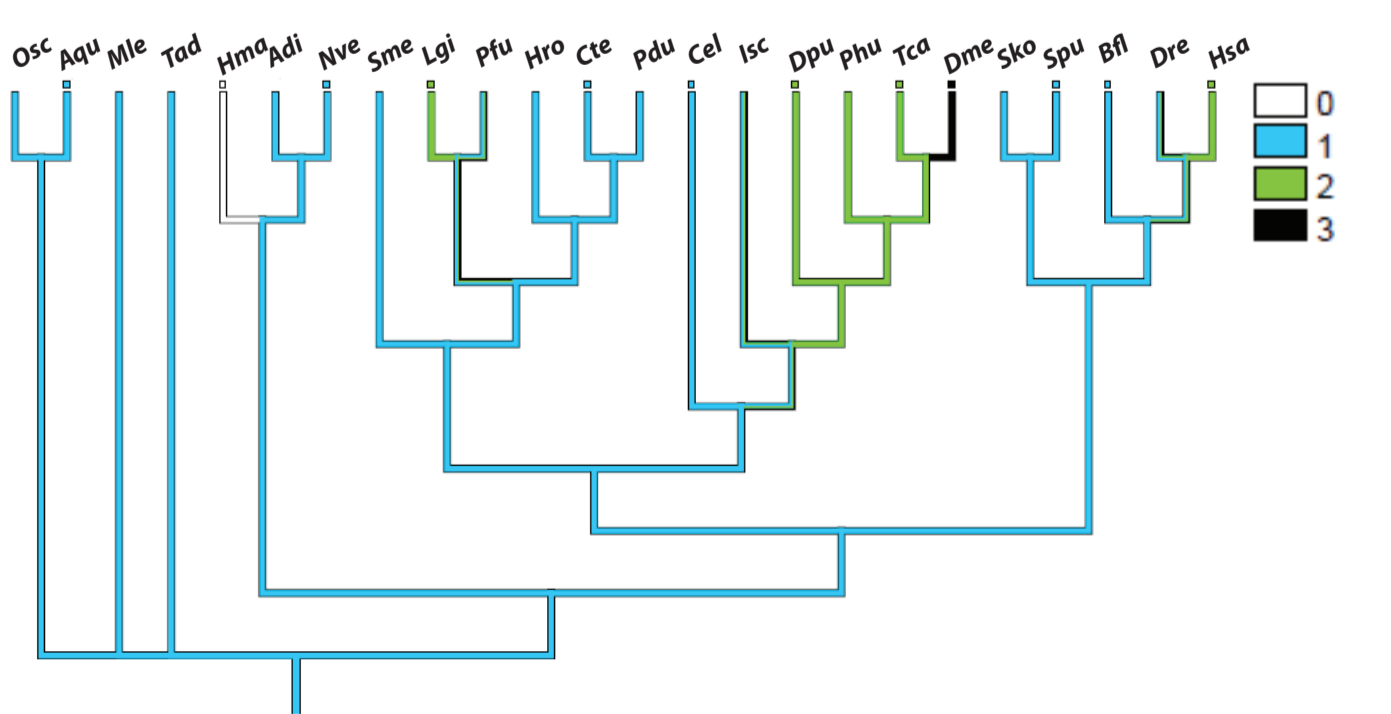

Supplement: Additional file 3: Figure S1 — Parsimony reconstruction analysis of character evolution based on a consensus Metazoan phylogenetic tree. The characters used in this analysis are the numbers of genes per basic helix-loop-helix (bHLH) family per species. Each character state is mentioned by a color code. Double-colored branches indicate non-determination of character state in the branch. The squares below taxon names give character state in the considered taxon; no square means unknown/missing data (in this case, character-state in the corresponding branch is optimized according to character-states in related taxa). A = Hes family: 1 to 22 Hes genes are present in the sampling dataset, the Urmetazoan presumably possessed one Hes gene and many duplications occurred. Gene loss is evidenced in one case. B = Hey family: 0 to 3 Hey genes are present in the sampling dataset, the Urmetazoan presumably possessed one Hey gene and 1 to 2 duplications occurred in the lineage leading to Capitella teleta and (Danio Rerio + Homo sapiens) clade only. Gene losses are evidenced in three cases. C = NeuroD family: 0 to 4 NeuroD genes are present in the sampling dataset, Urbilateria presumably possessed one NeuroD gene and duplications occurred in the lineage leading to (Danio rerio + Homo sapiens) clade. Gene loss is evidenced in one case. D = Clock family: 0 to 3 Clock genes are present in the sampling dataset, the Urmetazoan presumably possessed one Clock gene and one to two duplications occurred in several lineages. Gene loss is evidenced in one case. [file 2041-9139-5-29-S3.pdf]

# *Platynereis* larval development

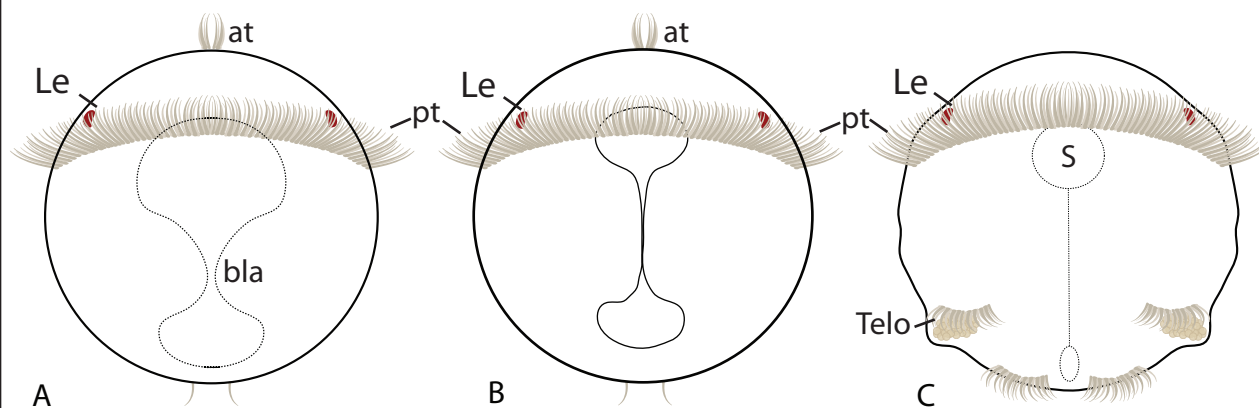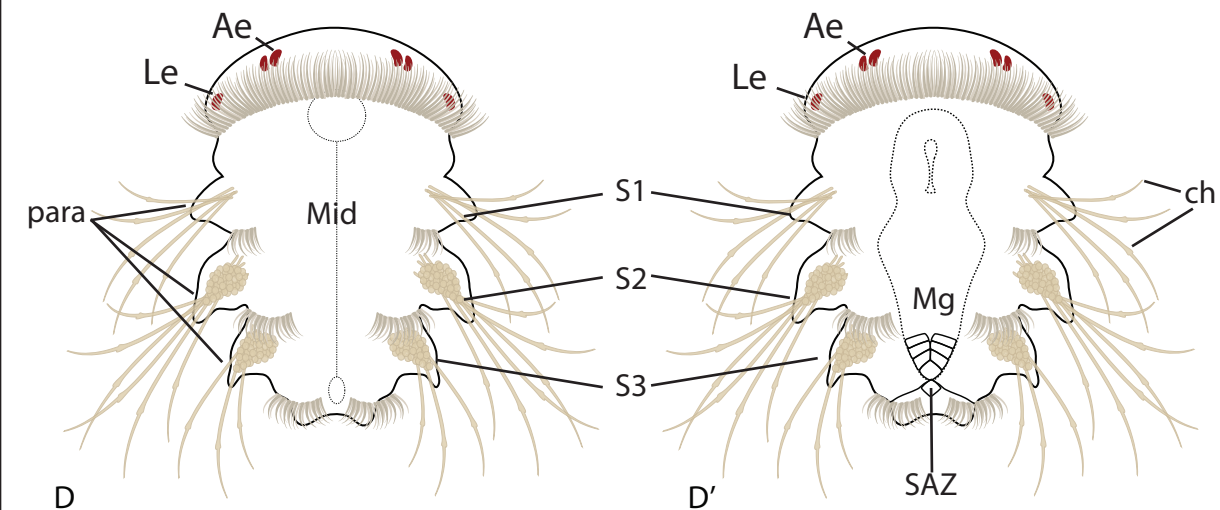

# Posterior elongation

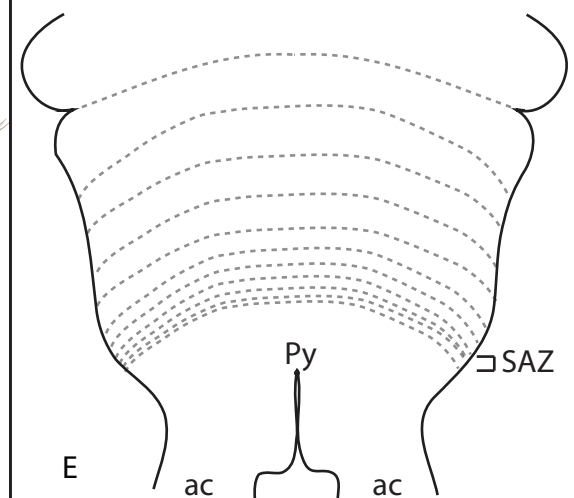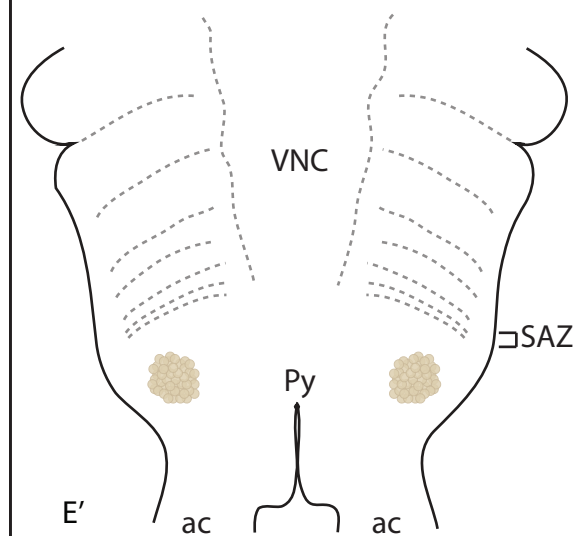

Supplement: Additional file 4: Figure S2 — Schematic drawings of Platynereis dumerilii general anatomy. Larval developmental stages studied as well as post-caudal regeneration posterior elongation process are shown. Those drawings are used in the main figures of the article for an easier comprehension of the expression patterns. A = 24 h post fertilization (hpf), ventral view; B = 33 hpf, ventral view, C = 48 hpf, ventral view; D = 72 hpf, ventral view (focusing on the neurectoderm); D = 72 hpf, deeper ventral view (focusing on internal structures such as the SAZ); E = post-caudal regeneration posterior elongation process, dorsal view; E’ = post-caudal regeneration posterior elongation process, ventral view. Ac = anal cirri; Ae = adult eye; At = apical tuft; bla = blastopore; ch = chaetae; Le = larval eye; Mg = midgut; Mid = midline; Para = parapodia; Pt = prototroch; Py = pygidium; S1 = 1st segment; S2 = 2nd segment; S3 = 3rd segment; S = stomodeum; SAZ = segment addition zone; Telo = telotroch; VNC = ventral nerve cord. [file 2041-9139-5-29-S4.pdf]

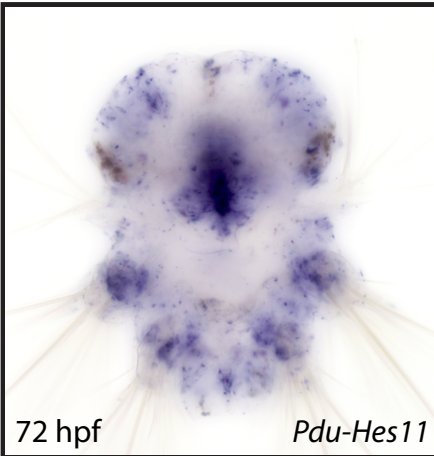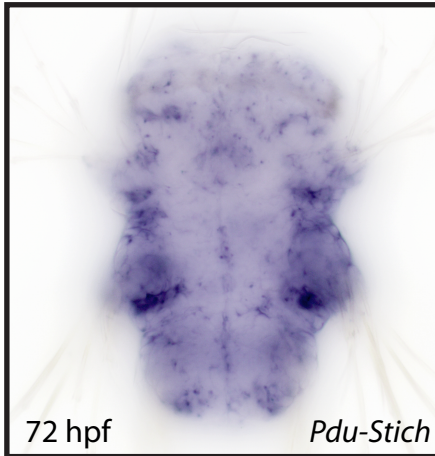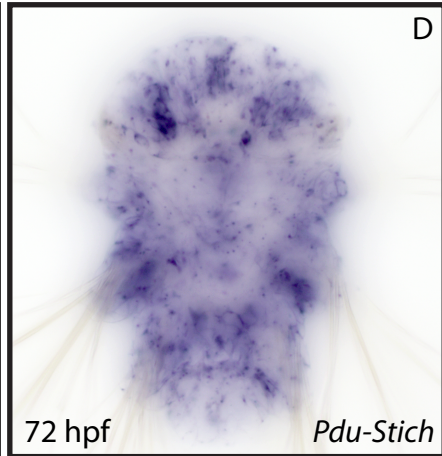

Supplement: Additional file 5: Figure S3 — Expression patterns of Pdu-Hes11 and Pdu-Stich at 72 h post fertilization (hpf). Whole-mount in situ hybridization (WMISH) for the 72hpf stage is shown. Pdu-Hes11 is expressed in various brain cells, stomodeum cells and mesodermal patches. In addition Pdu-Hes11+ cells are also observed in the segment addition zone (SAZ). Pdu-Stich is expressed in the midline cells, in various brain cells and mesodermal patches. Panels are mostly ventral views (anterior is up). A dorsal view (D) is also shown for Pdu-Stich. [file 2041-9139-5-29-S5.pdf]
